# EEG Correlates of Middle Eastern Music Improvisations on the *Ney* Instrument

**DOI:** 10.3389/fpsyg.2021.701761

**Published:** 2021-10-04

**Authors:** Mohammad Yaghmour, Padmakumari Sarada, Sarah Roach, Ibrahim Kadar, Zhivka Pesheva, Ali Chaari, Ghizlane Bendriss

**Affiliations:** ^1^Premedical Division, Weill Cornell Medicine Qatar, Doha, Qatar; ^2^Qatar Music Academy, Doha, Qatar

**Keywords:** improvisation, EEG, *Ney*, *Maqam*, prefrontal, cognition

## Abstract

The cognitive sciences have witnessed a growing interest in cognitive and neural basis of human creativity. Music improvisations constitute an ideal paradigm to study creativity, but the underlying cognitive processes remain poorly understood. In addition, studies on music improvisations using scales other than the major and minor chords are scarce. Middle Eastern Music is characterized by the additional use of microtones, resulting in a tonal–spatial system called *Maqam*. No EEG correlates have been proposed yet for the eight most commonly used *maqams*. The *Ney*, an end-blown flute that is popular and widely used in the Middle East was used by a professional musician to perform 24 improvisations at low, medium, and high tempos. Using the EMOTIV EPOC+, a 14-channel wireless EEG headset, brainwaves were recorded and quantified before and during improvisations. Pairwise comparisons were calculated using IBM-SPSS and a principal component analysis was used to evaluate the variability between the *maqams*. A significant increase of low frequency bands theta power and alpha power were observed at the frontal left and temporal left area as well as a significant increase in higher frequency bands beta-high bands and gamma at the right temporal and left parietal area. This study reveals the first EEG observations of the eight most commonly used *maqam* and is proposing EEG signatures for various *maqams*.

## Introduction

Human creativity has been the focus of thousands of studies and is still a topic of debate with considerable heterogeneity of evidence in brain research. Often, creativity is placed in the right hemisphere (Joseph, 1988; Hoppe, 1998; Demarin et al., 2016), but contradicting theories have been proposed to describe the underlying processes: (1) the dominance of right hemisphere activity (Joseph, 1988), (2) the low cortex activity (Carlsson et al., 2000; Starchenko et al., 2014), (3) the high neural connectivity (Razumnikova and Yashanina, 2014; Mayseless and Shamay-Tsoory, 2015), and (4) the prefrontal and frontal brain activation (Arden et al., 2010). Music improvisation refers to both the process and product of spontaneous creativity of music and constitutes a good model to study neural correlates of creative processes (Liu et al., 2012). Music improvisations, although spontaneous, are constructions resulting from successive decision-making processes. Nevertheless, research studies exploring the neural activity that underlies the creative process of music improvising remain scarce. Moreover, no study has explored the neural correlates of creating Middle Eastern Music, which involves the use of more than the major and minor scales.

Indeed, Middle Eastern Music is characterized by the use of *maqam*s (literally “place” and “position”), which are recognized as the system of melodic tunes used in traditional Middle Eastern Music from Turkey, Azerbaijan, Israel, Iran, and all Arab countries from Middle East to North Africa. These *maqams* are not exactly the equivalent of “scales” in Western Music and are characterized by defined tonal–spatial factors, while the rhythmic-temporal features are free (Touma, 1971). On the contrary, a waltz would have a fixed rhythmic-temporal organization, while the tonal–spatial component (the melody) is free. As a result, *maqams* differ in their intervals between the first few notes and are characterized by combinations of phrases that can include microtones such as quartertones, for example. Therefore, *maqams* resonate as various melodic tunes, that can be classified based on their characteristics into more than 50 families and subfamilies. The names given to each of these *maqams* are not subject to consensus, thus creating confusion in the literature. For this reason, it is always preferable to define the *maqam* by its set of intervals signatures as described in Table 1.

**TABLE 1:** Interval signatures of the eight *Maqam*s studied.

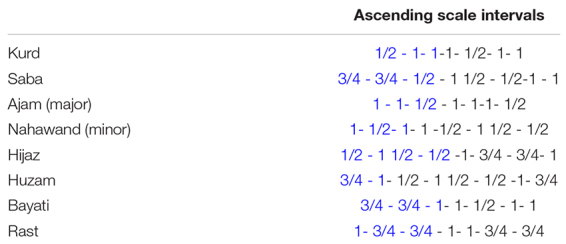

*By convention, the first few intervals, here noted in blue, give the name to the *maqam*.*

The music improviser builds the scales by using combinations of these families and subfamilies of *maqams*. In addition, associations of *maqams* to different emotional status are well rooted in Middle Eastern cultures (Kligman, 2001; Powers, 2005). Indeed, since the 9th century, philosophers and scientists such Al-Farab (870–950) have associated different *maqams* to different sets of emotions (Naroditskaya, 2009; Yöre, 2012). For example, the *maqam* called *Rast* is believed to trigger happiness, the *maqam* called *Saba*, is associated to sadness. Kligman (1993) has published a first trial of canonization of the *maqams* in the prayers of Syrian Jews in Brooklyn, New York, during their Shabbat morning service, where he described the association of *maqams* to various prayers. In his research, Kligman (2001) details how each maqam is said to convey a unique emotion and therefore preferable to use in specific prayers. In Turkey, research and clinical trials involving the use of *maqams* have been flourishing in the past years, and *maqams* are still believed to be associated to specific emotions (Özaslan et al., 2012; Bekiroğlu et al., 2013; Tumata, n.d.). Studies that support association of these maqams to specific emotions remain extremely scarce and none of them has explored the possibility of EEG-based emotion recognition. While Self-Assessment Mannequins have become a standard for emotion detection, a growing number of studies are using the development of machine learning algorithms and brain computer interfaces to propose novel methods for emotional assessments using brain signals, that are not be dependent on individual’s ability to express themselves or grasp of their mental state (Suhaimi et al., 2020; Torres et al., 2020). These studies have benefited from previous EEG explorations of neural correlates on Western Music and instruments. Lopata et al. (2017) compared improvisation-trained vs. non-improvisation-trained western musicians and showed an increase in right frontal upper alpha-band activity during more creative tasks such as improvisation, and their results suggested that creativity is probably a trainable mental state. Sasaki et al. (2019) showed in a study involving 14 male guitarists that improvisation over scale is characterized by an increase in power of theta, alpha, and beta bands in prefrontal and motor regions. Dikaya and Skirtach (2015) reported a distinguished EEG pattern in professional musicians during improvisation which was the predominant activation of the left-hemispheric cortical regions simultaneously with high interhemispheric integration in the high-frequency band.

Unfortunately, evidence on how humans perceive and process other modes of music such as the tonal–spatial system of *Maqams* is almost inexistent. Despite a recent study published in scientific reports by Teixeira Borges et al. (2019) that supported the role of scaling behavior of music in determining the emotions elicited, no study has yet explored and compared the neural correlates of different *maqams.* The present single subject study is the first exploration of EEG correlates to the eight most commonly used *maqams* and is believed to allow the expansion of this area of research to include Middle Eastern Music. Prior to this study, no research has focused on how *Ney* playing translates into EEG correlates.

One of the specificities of Middle Eastern Music is that it can include microtones such as quartertones, used in many *maqams*, but not all instrument can play those tones. This case study exploration relied on a performance using the *Ney* instrument, an end-blown flute, that has been played for more than 4500 years in the Middle East and is still an important component of today’s Middle Eastern musical ensemble. Playing the *Ney* requires a close coordination of mouth and jaw embouchure, lip contraction, diaphragm, and breathing control, in addition to fingering control. Moreover, similarly to the Clarinet, the *Ney* is known to be one of the most difficult Middle Eastern instruments to play, not only because it does not overblow in the octave, so almost every note has its own fingering and embouchure, but also because the *Ney* comes in thirteen different sizes, each one allowing the musician to play different ranges of octaves. These characteristics of the *Ney* instrument result in the necessity to coordinate multiple cognitive processes, including integration of sensory feedback, attention, working memory, decision making, movement, etc. (Ninaus, 2011).

*Ney* players and teachers often mention that playing the *Ney* is similar to singing and refer to the *Ney* as a continuum of their body into which they blow not only air, but also meaning and “words.” Although no research has previously focused on the cognitive demand of performing with this instrument, evidence supports the parallel of woodwind instruments and speech. Because woodwinds are hold at the continuity of the body and playing such instruments involves several parts of the body involved in singing (jaw, lips, tongue, mouth muscles, and breathing), it is expected that similarities are found between the cognitive demand of *Ney* and speech or singing (Zarate, 2013). Indeed, the musician’s lips function as a valve might for a woodwind instrument, just as the vocal folds need to be controlled to produce various resonances that are important for timbre, loudness, shape, and sharpness (Wolfe et al., 2009).

In addition to investigating *Ney* improvisations, this single-subject study is the first to explore EEG commonalities and differences among various music modes or *maqams* of Middle Eastern Music. The EEG is an excellent method to run a first exploration on this original topic, as it gives an overview of several cognitive processes in real time. Those cognitive processes include: working memory, retrieval, focus, concentration, relaxation, planning, decision making, motor planning, and arousal. Therefore, the aims of this short study are as following: (a) explore the EEG commonalities and specificities of *Ney* performance of various modes or *maqams*; (b) identify the EEG correlates of improvisations on *Ney* and comparing it with singing or spontaneous speech; (c) identify interesting patterns or signatures of *maqams* to explore further. This EEG-based study will set the ground for further explorations finding applications in musicology, music psychology, music performance, neurofeedback training, and music-based therapeutic interventions.

## Materials and Methods

### Materials

**FIGURE 1:**
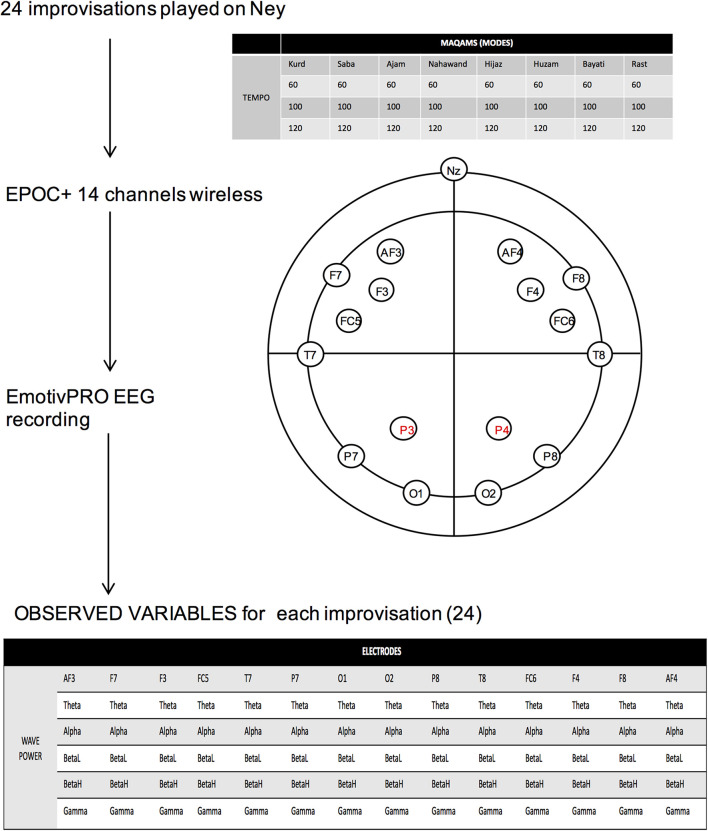
Study design. Detail of the 24 improvisations played and the 14 electrode positions, as well as the variables observed for each improvisation.

The EEG was recorded using the EMOTIV EPOCx headset (EMOTIV, San Francisco, CA, United States), a wireless headset that consists of 14 saline-based electrodes, recording at 14 sites according to the international 10–20 locations (AF3, F7, F3, FC5, T7, P7, O1, O2, P8, T8, FC6, F4, F8, and AF4) and two reference electrodes CMS/DRL at P3 and P4 (Figure 1). The headset also includes 9-axis motion sensors and detects head movements. The EMOTIV measurement system recorded in a sequential sampling at a sampling rate of 128 Hz, for a band width of 0.16–43 Hz. EEG signals were collected at a resting state with eyes closed, then relaxed with eyes opened. Signals includes digital notch filters at 50 and 60 Hz and built-in digital fifth order Sinc filter. Although the device is able to record performance metrics and facial expressions through its sensors, only EEG signals are analyzed and presented in this present paper. The data were wirelessly recorded using EMOTIV-PRO software running on the experimental computer.

### Experimental Set-Up and Recording

The subject is a healthy right-handed professional *Ney* player in the age range 30–40 years. Subject was comfortably sitting on a chair in a classroom with dim light and no other electronic than the recording computer. Subject was asked to play a total of 24 improvisations in a row, in the following melodic tunes: *Kurd, Saba, Bayati, Hijaz, Huzam, Ajam, Nahawand*, and *Rast.* Each melodic tune or *maqam is* defined by the intervals depicted in Table 1. The 24 tasks were listed in front of the performer. Each improvisation was played for 1 min at three different tempos: 60, 100, and 120 bpm, before moving on to the following *maqam*.

Another recording, referred to as the baseline (eyes opened, relaxed, and non-blowing status) was performed before starting the series of improvisations. The EEG was continuously recording throughout the experiment, which lasted 34 min and 32 s, including the setting and baseline recording as well as the time in between improvisations. To identify timestamps of beginning and end of each improvisation, markers were added by the experimenter using the keystroke marker function of the EMOTIV-PRO as described in their guidelines (Emotiv-Pro, 2013) and confirmed post-experimentally by visual observation of the timeline on the screen recording video of the entire experiment. A total of 25 min was included in the present analysis.

Improvisations were recorded using Audacity software and the EEG was recorded as described below. Figure 1 shows a summary of the design. The 24 improvisational audio clips can be found in Supplementary Files.

### Data Processing and Analysis

Using a band pass filtering system, the EMOTIV-PRO pre-processes and extracts the power spectra of the following frequency bands: theta (4–8 Hz); Alpha (8–12 Hz); low beta (12–16 Hz); high beta (16–25 Hz); and gamma (25–45 Hz). The EMOTIV-PRO provides the power spectra for all timestamps and electrode location in a csv file. All data recorded between improvisations were eliminated, and data were organized in an excel file based on the following variables: *maqam*, tempo, frequency band, and electrodes. Markers on timestamps were used to define beginning and end of each improvisation during the recording, allowing the calculation of mean power spectra in excel for the entire duration (1 min) of each improvisation. Data were uploaded into IBM-SPSS (version 26.0) to perform descriptive and statistical analysis. For the descriptive analysis, the mean powers of each frequency band per *maqam*, regardless of the tempo, as well as the mean powers of each frequency band per tempo, regardless of *maqam*, were compared. In addition, the mean powers of each electrode separately were compared between each *maqam* regardless of tempo, and between each tempo regardless of *maqam*.

This multivariate study used a two-way analysis of variance (ANOVA) to test the hypotheses. The outcome variable was the power, which is quantitative, while all the independent variables were qualitative such as *maqam* (8 levels), frequency bands (5 levels), tempo (4 levels), and electrode (14 levels). The statistical analysis was done using SPSS to see the main effects as well as the interaction effects of the independent variables on the outcome variable. The interaction term reveals whether the effect of one independent variable on the outcome variable is the same for all values of the other independent variable. *Post hoc* tests were administered for the pairwise comparisons. Separate plots were prepared for main effects and interaction effects. The assumption of homogeneity was tested using Levene’s test of homogeneity. The confidence level was set at 95%. Research questions were answered here using the two-way ANOVA technique. Heatmaps produced on excel were computed for each frequency band separately and included minimum and maximum values from the mean power spectra for all *maqams*. To better visualize eventually asymmetry, cells were then reorganized into right/left and frontal/occipital for each *maqam*.

Using IBM-SPSS, Kaiser–Meyer–Olkin, and Bartlett’s tests were used to measure sampling adequacy for principal component analysis (PCA) analysis. Eigen values and scree plots were computed to find the number of components that can be used. Four components were used for PCA analysis of the eight *maqams*. Detailed output of the analysis is available in Supplementary File 3.

## Results

### Changes in Slow Oscillations at the Left Frontal and Left Temporal Area

**FIGURE 2:**
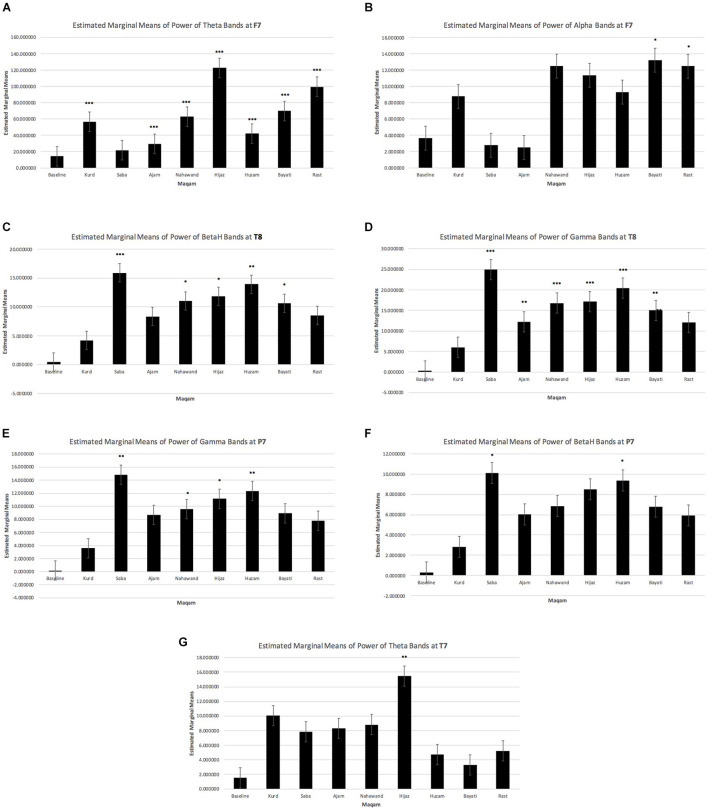
Left fronto-temporal increase of low frequency bands and parietal left and temporal right increase of high frequency bands during improvisations for each maqam as compared to the baseline. **(A)** Theta bands at F7. **(B)** Alpha bands at F7. **(C)** Beta high bands at T8. **(D)** Gamma bands at T8. **(E)** Gamma bands at P7. **(F)** Beta high bands at P7. **(G)** Theta bands at T7.

For each *maqam*, a different improvisation was performed at 60, 100, and 120 bpm, which allowed the calculation of a mean of spectral powers for each electrode and for each *maqam*, compared to the baseline (non-blowing and non-improvising resting state). Figure 2 suggests a highly significant increase of theta bands at the frontal left area F7 for almost all maqams (*p*-value < 0.001), except for the maqam Saba. The details of the means and *p*-values for all electrodes are available in Supplementary File 1.

Because these means were performed between different tempos, they reflect the effect of the maqam regardless of the tempo. There seem to be some topographical differences as well as power differences as the tempo increases. However, the effect of the tempo was not tested statistically, since only one improvisation per maqam and per tempo was performed. A Supplementary Figure that details the mean power of bands obtained at each tempo is available in Supplementary File 3.

Observations suggest some specificities of improvising on certain maqams. A highly significant increase of theta bands compared to the baseline is observed for the *maqam Ajam* in the frontal left area F3 (*p*-value = 0.000) (Supplementary Table 1) and the *maqam Hijaz* at the left temporal area T7 (*p*-value = 0.003) (Supplementary Table 1). In addition, we also observe at F7 a significant increase of alpha bands for the *maqam Bayati* at the left frontal area F7 (*p*-value = 0.039) and the *maqam Rast* (*p*-value = 0.05) (Table 1).

### Changes in Fast Oscillations at the Left Parietal and Right Temporal Area

At the left parietal area P7, the two *maqams Saba and Huzam* showed significant increase of beta-high bands (*p*-value = 0.034, *p*-value = 0.050, respectively) and gamma bands (*p*-value = 0.002, *p*-value = 0.009, respectively) (Supplementary Table 1). Two other maqams, *Nahawand and Hijaz*, showed a significant increase at P7, but only for gamma bands (*p*-value = 0.043, *p*-value = 0.018) (Supplementary Table 1).

At the right temporal area T8, almost all *maqams* were characterized by a highly significant increase in gamma bands (*p*-value = 0.000). The only maqam that did not show a significant increase in gamma at T8 was the *maqam Kurd*. For beta-high bands, the increase was significant at T8 for the maqam Saba (*p*-value = 0.001), Nawahand (*p*-value = 0.023), Hijaz (*p*-value = 0.014), Huzam (*p*-value = 0.004), and Bayati (*p*-value = 0.028), but not significant for Kurd, Ajam, and Rast (Supplementary Table 1).

Data suggest that, at the contrary to beta-high bands, the activity of beta-low bands is increased not only in T8 and P7, but also in F7. However, the pairwise comparisons for beta-low were not significant (Supplementary File 4).

### Improvisations Done on Different Maqams Induce Different EEG Signatures

Three different improvisations were played for each maqam, at three different tempos and the mean power spectra for each maqam were used to perform pairwise comparisons with the baseline and between all *maqams*. Table 2 summarizes the significant changes observed in all maqams as compared to the baseline and suggests the existence of *maqam* EEG signatures. Improvisations on the *maqams saba* and *huzam* both showed significant increase of beta high and gamma bands at P7 and T8. However, the maqam *huzam* differed by a significant increase of theta at F7. In addition, improvisations made on maqam *saba* produced significantly higher power spectra of gamma bands than all other *maqams* (*p*-value < 0.001) (Table 2) not only at locations P7 and T8, but also at locations T7, P8, and O1 (Supplementary Figure 4).

**TABLE 2:** Proposed EEG signatures for improvisations at eight commonly used *maqams*.

	**Significant *p*-values (compared to baseline)**							
**Improvisation mode**	**Theta (4–8 Hz)**			**Alpha (8–12 Hz)**	**Beta high (16–25 Hz)**		**Gamma (25–45 Hz)**	
	**F7**	**F3**	**T7**	**F7**	**P7**	**T8**	**P7**	**T8**
Kurd	0.000	–	–	–	–	–	–	–
Saba	–	–	–	–	0.034	0.001	0.002	0.000
Ajam	0.001	0.000	–	–	–	–	–	0.010
Nahawand	0.000	–	–	–	–	0.023	0.043	0.000
Hijaz	0.000	–	0.003	–	–	0.014	0.018	0.000
Huzam	0.000	–	–	–	0.050	0.004	0.009	0.000
Bayati	0.000	–	–	0.039	–	0.028	–	0.002
Rast	0.000	–	–	0.053	–	–	–	0.010

The *maqam Hijaz* is the only *maqam* studied that includes an interval of 1.5 tone and also happens to be the only maqam on which improvisations induced a significant increase of theta at the left temporal region T7 (Table 2). Improvisations done on *Hijaz* showed the highest theta activity at almost all brain locations (Supplementary Figure 4). The maqam *hijaz* depicts significant changes at similar topographic area to improvisations done on maqam *nahawand*, with significant increase of theta bands at F7, beta-high at T8, and gamma at P7 and T8.

The improvisations on *Kurd* showed the lowest power of gamma across all locations and whatever the tempo, did not increase in T8 as in all other *maqams* (Supplementary Figure 4). The pairwise comparisons confirmed that the gamma activity of improvisations on *Kurd* in T8 are not significantly different from the baseline and significantly lower than on all other *maqams* (*p*-value < 0.001). The only location where significant changes were observed for improvisations on *maqam kurd* was F7 with an increase in theta bands (Table 2).

**FIGURE 3:**
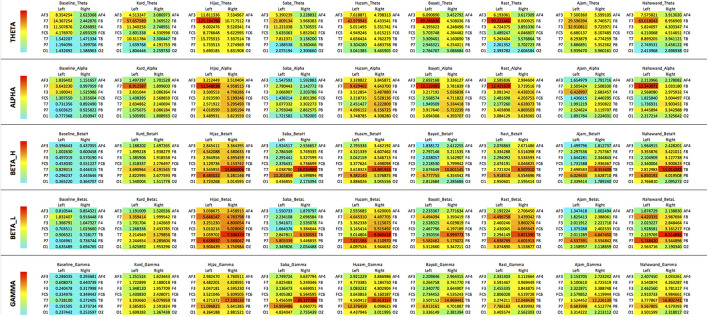
Heatmaps of power spectra of frequency bands at 14 electrode sites for each *maqam*. Heatmap were computed for each frequency band type across all *maqams.* Power spectra are organized in a way to simulate scalp map (frontal on top, occipital on bottom, left, and right hemispheres).

Figure 3 depicts heatmaps for all the *maqams* tested. The heatmaps are organized in such a way to simulate scalp maps and show the asymmetry of brain activation for each band type at the frontal, temporal, parietal, and occipital regions of the brain. Although these do not account for the significance of the changes, these maps propose a hypothetical patterns of brainwave activation per *maqam*, to be explored further.

**FIGURE 4:**
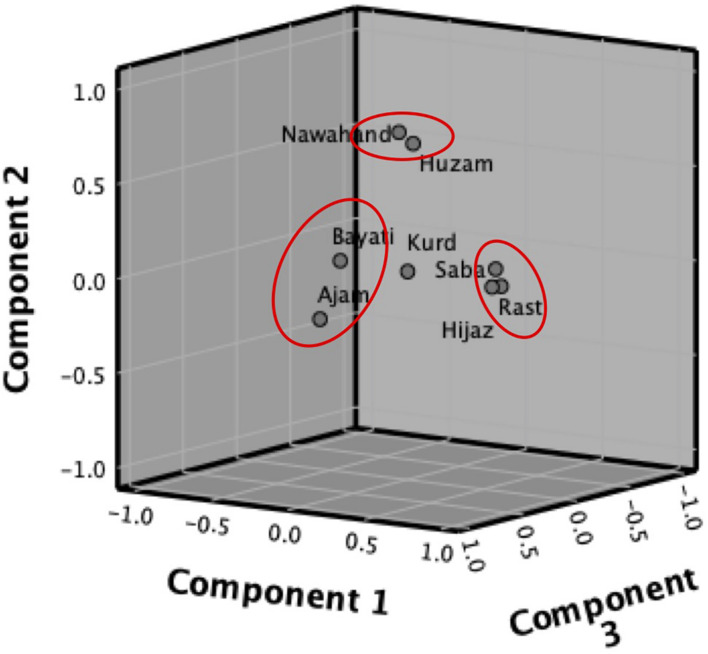
Principal component analysis plot of the eight *maqam* commonly used in Middle Eastern Music. Detailed output available in Supplementary File 5.

In addition, a principal component analysis was performed on the eight maqams to explore any eventual proximity between the *maqams*. The Bartlett’s test was significant (*p*-value < 0.05) and concluded that our data have enough variance to be partitioned using factor analysis. Figure 4 depicts the component plot in rotated space and suggests the presence of three clusters, with the *maqam kurd* being the closest to zero. Figure 4 suggests the presence of three clusters of improvisations around the *maqam kurd*: (1) *bayati* and ajam, (2) *saba*, *rast*, and *hijaz*, and (3) *nahawand* and *huzam*. These clusters will be interpreted and discussed in the following section.

## Discussion

### EEG Correlates of Improvisations on the *Ney* Instrument

Musical improvisation is a complex musical behavior that captured attention of a growing number of scientists. This single subject case aimed at exploring human creativity by exploring improvisations *via* the prism of Middle Eastern Music and using the *Ney* flute, which were not explored before. In this single case study, we observed significant changes in the powers of the low frequency bands (theta and alpha) in the left frontal and left temporal areas −F7, F3, and T7. In addition, significant increases in the powers of the higher frequency bands (beta-high and gamma) were observed in the left parietal and right temporal areas, P7 and T8. These results align with previous studies done on western music improvisation and using other instruments. Indeed, Dikaya and Skirtach (2015) showed in a cohort of 136 musicians, amateur, and professionals, that professional musicians were distinguished by a predominant activation of the left hemisphere, with a simultaneous integration between both hemispheres in the higher frequency bands, which is similar to what we have observed in this experiment done on a professional musician. Other studies showed this increase of the EEG spectral power at the prefrontal cortical area, when playing guitar (Sasaki et al., 2019), rock music (Tachibana et al., 2019), and piano (Pinho et al., 2014). The specific increase of frontal theta is facilitated by emotions, concentration, and mental tasks (Aftanas and Golocheikine, 2001; Marcuse et al., 2016; Katahira et al., 2018). While EEG allows an extremely good temporal resolution, it does not provide a good spatial resolution and as a result, studies using EEG do not inform on accurate location of the observed data. Some have used other techniques to explore improvisation cognitive demand such as Limb and Braun (2008), who used fMRI on jazz pianists improvising novel melodies using pre-existing chord patterns (equivalent to our experiment with the *maqams*) and highlighted an activation in lateral and prefrontal area. In a study of Faber (2014), an exploration involving 32 channels during improvisations in various settings, a strong activity of the frontal and central–temporal regions were also observed suggesting improvised music is a communicative medium. Itthipuripat et al. (2013) showed that frontal theta constitutes a signature of a successful working memory manipulation, as theta bands are also known to facilitate the encoding of temporary episodic memories into long-term memory (Baddeley, 2003; Itthipuripat et al., 2013). Interestingly, at the contrary to all other *maqams*, improvisations done on the maqam *Saba* did not induce significant increase of theta bands at the F7 frontal area, suggesting the possible use of different cognitive processes for improvisations done on this *maqam*.

The ability to improvise is one of the highest levels of musical achievement, as it requires from the improviser to master the music language necessary to spontaneously compose original music. The cognitive processes underlying improvisations have been compared to spontaneous speech (Peretz et al., 2015). Therefore, it is thought to be a powerful mean to express oneself and communicate with others. Nevertheless, these processes are still poorly understood. The data obtained through this single subject study corroborate the existing studies showing an increase in the EEG spectral power at the pre-frontal area (Sasaki et al., 2019), suggesting a strong role for the region F7 or left frontal area of the brain, involved in controlling language-related movement and executive functions such as planning, organizing, and self-monitoring. We have found that most improvisations in this present study resulted in significant increase of beta-high and gamma activity at parietal left P7 and temporal right T8 area. Wan et al. (2014) explored the EEG signals of a pianist on improvisations using Western Music scales have also suggested that frontal, parietal, and temporal regions play a key role in differentiating improvisations from playing composed music. Interestingly, they noted the strong involvement of T8 but not P7 (Wan et al., 2014). While T8 represents the right temporal lobe, close to the amygdala and hippocampus and involved in auditory processing and music appreciation (Stewart et al., 2006), P7 is involved in logical or verbal understanding, word recognition during auditory processing. We emitted the hypothesis that EEG signals on woodwind instrument such as the *Ney* would be similar to those during spontaneous speech. The strong involvement of F7, T7, and P7 that we noted for *Ney* improvisations were also spotted by Chengaiyan et al. (2020) in a study of speech imagery (or imagining of speaking), where they observed that left frontal and left temporal electrodes (where T5 correspond to our P7) were activated for speech and speech imagery processes (Chengaiyan et al., 2020).

### EEG Signatures of *maqams* in Middle Eastern Music

Musicology research studies on *maqams* are extremely limited and almost exclusively run in Turkey and Israel. However, no study has explored yet the neural correlates of these *maqams* in the same way studies have explored the EEG correlates of major and minor chords (Petsche et al., 1996; Virtala et al., 2013). When having a closer look at the EEG signals elicited by improvisations at each maqam, we observed that each *maqam* was characterized by a topographically unique combination of significant electroencephalographic changes, suggesting the existence of what we would call *maqam* EEG signatures, as presented in Table 2. Jenni et al. (2017) explored processing of western tonal music major and minor EEG signals and observed an increased activity of higher frequency bands for the minor scale. Interestingly, in the present study, we observed the same pattern between improvisations on *Nahawand* (minor) and the those on *Ajam* (major). But because Middle Eastern Music is also using other tones than the whole tone and semi-tone, a greater number of scales is possible yielding the maqams families studied. It is therefore of great importance that we understand the interactions between the *maqams*, in order to build future studies on emotional correlates of these *maqams*. Therefore, we have opted for a principal component analysis to extract eventual clusters that could help us understand which feature in these improvisations seem to play a key role. The principal component analysis suggested the presence of three clusters of improvisations around the *maqam Kurd*: (1) *Bayati* and *Ajam*, (2) *Saba*, *Rast*, and *Hijaz*, and (3) *Nahawand* and *Huzam*. The main challenge in studying the *maqam* system resides in the terminology used, as these names actually refer to the first sets of intervals described in Table 1.

The term *jins* (or plural *ajnas*) refers to the building blocks of a *maqam* scale, which always has a lower and an upper *jins*. By convention, *maqams* are classified based on their lower *jins*, and the first note of the second *jins* is called the dominant and is the second most important note after the tonic.

By comparing Table 1 and the PCA plot, we understand that the clusters we see could correspond to the sets of intervals they have in common. Indeed, *Ajam* – characterized by the intervals 1-1-1/2 – is also present in within the unfolding of the *Bayati* scale 3/4 - 3/4 - 1- 1- 1/2 - 1- 1. The maqam *Saba* includes some *Kurd* (1/2 - 1- 1) and some *Hijaz* (1/2 - 1 1/2 - 1/2), while the *maqam Hijaz* includes some *Rast* (1- 3/4 - 3/4), possibly explaining the proximity of these three *maqams* on the PCA. As for the *Kurd* set of intervals 1/2 - 1- 1 is retrieved in *Kurd*, *Saba*, *Ajam*, *Bayati*, *Nahawand* and could explain why this particular *maqam* is not fitting in any of the clusters.

These interactions between *maqams* are well known by professional musicians and structural proximity of these scales are used to create improvisations that increase in complexity by mixing closely related *maqams*. Dikaya and Skirtach (2015) suggested that high-frequency coherent connections increased with the level of difficulty of the musical improvisation.

This result is important because it highlights the importance of considering intervals, tones, and microtones, in studies on processing of music and emotional correlates.

### Implications for Studies on EEG-Based Detection of Emotions

Gu (2014) in her book, “Cultural history of Arabic Language,” mentioned the emotions that are commonly associated to each of the presently studied *maqam*. *Kurd* evoking freedom, romance, and gentleness; *Saba* evoking sadness or pain; *Ajam* evoking strength; *Nahawand* evoking drama and emotional extremes; *Hijaz* evoking desert, solitude, and enchantment; *Huzam* evoking old days; *Bayati* evoking femineity, joy, and vitality; and *Rast* evoking pride and power. Although there is no consensus among musicologists on what the mood each *maqam* is associated with, it is surprising to see to which extent these various mood-*maqam* associations have been passed on throughout history without any scientific methodology or validated emotional assessment to support these claims (Kligman, 2001; Powers, 2005; Gu, 2014; Marks, 2019).

EEG signals play an important role in research on human emotions, which in turn are involved in cognitive processes such as memory, learning, and decision-making (Zhang et al., 2020). There are converging evidence from the literature that gamma bands, in addition to being associated with focus and concentration, are associated with negative emotions such as sadness and worry (Oathes et al., 2008; Yang et al., 2020). A recent study using functional network analysis by Yang et al. (2020) exposed native Chinese individuals to 180 pictures selected from the Chinese Affective Picture System (CAPS) (Lu et al., 2005) and recorded their EEG responses as well as their self-assessment Manikin rating scales (Morris, 1995). While no significant difference in brain network were found at low frequency bands, significant differences were observed between positive and negative emotions In the high gamma bands. They concluded on the existence of neural signatures for emotional states in the high gamma bands, particularly against negative stimuli (Yang et al., 2020).

Interestingly, the *Saba maqam is* consistently called by musicians of the Middle East the “*sad maqam*” and is also depicting the highest gamma spectral power in this case study. This result represents the first piece of EEG evidence that could eventually support the historical claim that *Saba maqam* is associated to negative emotions. However, since we did not perform any emotional assessment during the experiment, further studies are needed to conclude on this. In addition, as this results from a single subject study, more explorations on larger and culturally diverse samples need to be performed.

*The Hijaz maqam* showed the highest theta activity across all brain locations. The theta bands have been shown to increase during sleep, deep meditation, and spiritual awareness. Some have described the *Hijaz maqam* as being “snake charming music.” Powers (2005, p. 9) writes that “this *Maqam* is associated with the lonely treks of the camel caravans and with fascination and enchantment.” Lee et al. (2018) have reviewed the neural signals underlying several meditation practices including focused attention, open-monitoring, transcendental attention and loving kindness meditation. It is only during focused attention that a significant increase of theta was observed across both anterior and posterior parts of the brain. While further characterization of the oscillatory activities during improvisation on *Hijaz* are necessary, the present data suggest similarities between improvisations on *Hijaz* and focused attention practices.

Music and perception have been substantially researched in the field of music psychology. Through existing neuronal measuring methodologies (EEG, fMRI, and MEG), studies shed light on brain functionality and mechanisms involved when passively listening or playing an instrument (Limb and Braun, 2008; Virtala et al., 2013; Almudena et al., 2021). It is generally agreed that music stimulates a combination of different processes including short-term memory, the nature of different emotions produced by music, concentrations, pleasure and non-pleasure, and self-reflection (Almudena et al., 2021). Studies of Almudena et al. (2021) and Virtala et al. (2013) support that different mechanisms are involved in the perception of major vs. minor, and consonant vs. dissonance chords in infants, adults and school-age children, which correlate with findings in the present study. Furthermore, Limb’s study (Limb and Braun, 2008) tries to break the code of spontaneous music performance, with the assumption that this creative music process is predicative on novel combination of ordinary mental processes. It was hypothesized that short term memory would be associated with hierarchical top-down subtle changes in other systems, such as sensorimotor area and limbic structures used to regulate memory and emotional tone. The study suggests that the prefrontal cortex is of critical importance for processes which include self-reflection, and sensory processes as integral component.

To conclude, this present case explored the power of low and high frequency bands across 14 cortical locations during Middle Eastern Music improvisations played using the *Ney* instrument using the tonal–spatial system of *Maqams*. This case provides further support to the already published studies on the important role during musical improvisations of the left hemisphere with the significant increase of the low frequency bands at the frontal and temporal left area, as well as the more integrated activity in both hemispheres at higher frequency bands.

In addition, this case introduces, for the first time in neuromusicology, the question of EEG *Maqam* signatures, where signatures found seems to follow the maqam’s intervals signatures, supporting the necessity of referring to *Maqams* by their intervals rather than their names.

Single case studies are being used by many across multiple sessions to obtain consistent results using brain activity measurements (Farrugia et al., 2021).

Finally, this case’s results can be used as ground study to design further studies, including: (1) establishing the cognitive demand for each mode or *maqam* on professional’s vs. amateur musicians or improvisation vs. composed music, (2) exploring the listener’s and the performer’s perception of intended emotions by using the present recordings of various *maqams* and combination of self-assessment mannequin and EEG-based emotion detection, and (3) increasing the sample size in order to confirm the proposed correlates and explore the possibilities of neurofeedback training to improve performance on every *maqam*.

## Data Availability Statement

The raw data supporting the conclusions of this article will be made available by the authors, without undue reservation.

## Ethics Statement

Ethical review and approval was not required for the study on human participants in accordance with the local legislation and institutional requirements. The patients/participants provided their written informed consent to participate in this study.

## Author Contributions

GB worked on the conceptualization, methodology, analysis, investigation, interpretation and writing, and supervised all the work. MY worked on the methodology, ran the investigation, managed software processing of data, did the analysis, and wrote parts of the manuscript. PS worked on the methodology, managed software processing of data, did the analysis, and wrote parts of the manuscript. SR participated in running the investigation, writing, reviewing, and editing. IK ran the investigation, participated in interpretation, writing, reviewing, and editing. ZP and AC participated in writing, reviewing, and editing. All authors have read and agreed to the published version of the manuscript.

## Conflict of Interest

The authors declare that the research was conducted in the absence of any commercial or financial relationships that could be construed as a potential conflict of interest.

## Publisher’s Note

All claims expressed in this article are solely those of the authors and do not necessarily represent those of their affiliated organizations, or those of the publisher, the editors and the reviewers. Any product that may be evaluated in this article, or claim that may be made by its manufacturer, is not guaranteed or endorsed by the publisher.

## Supplementary Material

Supplementary File 1 Mean power spectra of theta, alpha, beta-low, beta-high, and gamma at each of the 14 electrodes: pairwise comparisons between baseline and each *maqam.*

Supplementary File 2 Mean power spectra of theta, alpha, beta-low, beta-high, and gamma bands at the 14 electrode sites and for all maqams.

Supplementary File 3 Detail of the power spectra of theta, alpha, beta-low, beta-high, and gamma bands at the 3 different tempos tested, at the 14 electrode sites and for all maqams.

Supplementary File 4 Mean power spectra of theta, alpha, beta-low, beta-high, and gamma bands at the 14 electrodes organized by *maqam*.

Supplementary File 5 Detailed output of principal component analysis.

Supplementary Audios Improvisations played are available in Supplementary Files.
