# Supplementary material for: EEG Correlates of Middle Eastern Music Improvisations on the Ney Instrument
Source: Front Psychol. 2021 Oct 4;12:701761. doi: 10.3389/fpsyg.2021.701761 (PMC8520950; doi:10.3389/fpsyg.2021.701761)
Supplement: Supplementary File 2 — Mean power spectra of theta, alpha, beta-low, beta-high, and gamma bands at the 14 electrode sites and for all maqams. [file Data_Sheet_2.PDF]

Left

AF3

Wave	Electrode	(I)	(J)	Mean Difference (I-J)	Std. Error	Sig.[b]	95% Confidence Interval for Difference[b] Lower Bound	Upper Bound
Theta	AF3	Baseline	Kurd	3.838	4.642	0.408	-5.260	12.936
			Saba	2.989	4.631	0.519	-6.087	12.065
			Ajam	0.850	4.633	0.854	-8.232	9.931
			Nahawand	0.776	4.634	0.867	-8.306	9.859
			Hijaz	-3.379	4.630	0.466	-12.452	5.695
			Huzam	0.698	4.628	0.880	-8.372	9.768
			Bayati	2.261	4.636	0.626	-6.825	11.348
			Rast	2.164	4.559	0.635	-6.772	11.099
Alpha	AF3	Baseline	Kurd	0.334	4.642	0.943	-8.765	9.432
			Saba	0.287	4.631	0.951	-8.789	9.363
			Ajam	0.172	4.633	0.970	-8.910	9.253
			Nahawand	-0.391	4.634	0.933	-9.474	8.692
			Hijaz	-1.377	4.630	0.766	-10.450	7.697
			Huzam	-1.516	4.628	0.743	-10.586	7.554
			Bayati	-0.876	4.636	0.850	-9.962	8.211
			Rast	-0.758	4.559	0.868	-9.694	8.178
Beta-L	AF3	Baseline	Kurd	-0.377	4.642	0.935	-9.475	8.722
			Saba	-0.731	4.631	0.875	-9.807	8.345
			Ajam	-0.536	4.633	0.908	-9.618	8.545
			Nahawand	-1.070	4.634	0.817	-10.152	8.013
			Hijaz	-2.225	4.630	0.631	-11.299	6.848
			Huzam	-2.142	4.628	0.643	-11.212	6.928
			Bayati	-1.429	4.636	0.758	-10.516	7.657
			Rast	-1.380	4.559	0.762	-10.316	7.555
Beta-H	AF3	Baseline	Kurd	-0.788	4.642	0.865	-9.886	8.310
			Saba	-1.518	4.631	0.743	-10.594	7.558
			Ajam	-1.103	4.633	0.812	-10.184	7.978
			Nahawand	-1.571	4.634	0.735	-10.654	7.512
			Hijaz	-2.450	4.630	0.597	-11.524	6.624
			Huzam	-2.412	4.628	0.602	-11.482	6.658
			Bayati	-1.444	4.636	0.755	-10.531	7.642
			Rast	-1.679	4.559	0.713	-10.615	7.257
Gamma	AF3	Baseline	Kurd	-0.963	4.642	0.836	-10.061	8.135
			Saba	-2.445	4.631	0.598	-11.520	6.631
			Ajam	-1.867	4.633	0.687	-10.948	7.214
			Nahawand	-2.123	4.634	0.647	-11.205	6.960
			Hijaz	-2.700	4.630	0.560	-11.774	6.374
			Huzam	-2.643	4.628	0.568	-11.712	6.427
			Bayati	-1.927	4.636	0.678	-11.013	7.160

			Rast	-2.043	4.559	0.654	-10.979	6.893

F7

Wave	Electrode	(I)	(J)	Mean Difference (I-J)	Std. Error	Sig.[b]	95% Confidence Interval for Difference[b] Lower Bound	Upper Bound
Theta	F7	Baseline	Kurd	-42.385*	4.642	0.000	-51.483	-33.287
			Saba	-7.524	4.631	0.104	-16.600	1.552
			Ajam	-15.234*	4.633	0.001	-24.316	-6.153
			Nahawand	-48.545*	4.634	0.000	-57.628	-39.462
			Hijaz	-108.154*	4.630	0.000	-117.228	-99.080
			Huzam	-27.830*	4.628	0.000	-36.900	-18.760
			Bayati	-55.474*	4.636	0.000	-64.561	-46.388
			Rast	-85.306*	4.559	0.000	-94.242	-76.371
Alpha	F7	Baseline	Kurd	-5.114	4.642	0.271	-14.212	3.984
			Saba	0.859	4.631	0.853	-8.217	9.935
			Ajam	1.138	4.633	0.806	-7.943	10.219
			Nahawand	-8.837	4.634	0.057	-17.919	0.246
			Hijaz	-7.711	4.630	0.096	-16.784	1.363
			Huzam	-5.657	4.628	0.222	-14.727	3.413
			Bayati	-9.568*	4.636	0.039	-18.655	-0.482
			Rast	-8.826	4.559	0.053	-17.761	0.110
Beta-L	F7	Baseline	Kurd	-1.476	4.642	0.751	-10.574	7.622
			Saba	-0.390	4.631	0.933	-9.466	8.686
			Ajam	0.007	4.633	0.999	-9.074	9.089
			Nahawand	-2.584	4.634	0.577	-11.667	6.499
			Hijaz	-3.761	4.630	0.417	-12.834	5.313
			Huzam	-2.809	4.628	0.544	-11.879	6.261
			Bayati	-2.689	4.636	0.562	-11.776	6.397
			Rast	-2.674	4.559	0.557	-11.610	6.261
Beta-H	F7	Baseline	Kurd	-0.888	4.642	0.848	-9.986	8.210
			Saba	-1.766	4.631	0.703	-10.842	7.310
			Ajam	-1.294	4.633	0.780	-10.376	7.787
			Nahawand	-2.355	4.634	0.611	-11.438	6.728
			Hijaz	-3.524	4.630	0.447	-12.598	5.550
			Huzam	-3.335	4.628	0.471	-12.405	5.735
			Bayati	-1.800	4.636	0.698	-10.887	7.286
			Rast	-2.332	4.559	0.609	-11.268	6.604
mma	F7	Baseline	Kurd	-1.111	4.642	0.811	-10.209	7.987
			Saba	-3.190	4.631	0.491	-12.266	5.885
			Ajam	-2.491	4.633	0.591	-11.573	6.590
			Nahawand	-2.935	4.634	0.526	-12.018	6.148
			Hijaz	-3.871	4.630	0.403	-12.945	5.202

Wave	Electrode	(I)	(J)	Mean Difference (I-J)	Std. Error	Sig.	Lower Bound	Upper Bound
Ga			Huzam	-4.172	4.628	0.367	-13.242	4.898
			Bayati	-2.660	4.636	0.566	-11.747	6.426
			Rast	-2.980	4.559	0.513	-11.916	5.955

F3

Wave	Electrode	(I)	(J)	Mean Difference (I-J)	Std. Error	Sig.[b]	95% Confidence Interval for Difference[b] Lower Bound	Upper Bound
Theta	F3	Baseline	Kurd	0.187	4.642	0.968	-8.911	9.285
			Saba	0.309	4.631	0.947	-8.767	9.385
			Ajam	-21.235*	4.633	0.000	-30.317	-12.154
			Nahawand	5.691	4.634	0.219	-3.391	14.774
			Hijaz	2.581	4.630	0.577	-6.493	11.655
			Huzam	6.455	4.628	0.163	-2.615	15.525
			Bayati	7.097	4.636	0.126	-1.989	16.184
			Rast	6.782	4.559	0.137	-2.153	15.718
Alpha	F3	Baseline	Kurd	0.329	4.642	0.944	-8.769	9.427
			Saba	0.105	4.631	0.982	-8.971	9.180
			Ajam	-3.241	4.633	0.484	-12.322	5.841
			Nahawand	0.591	4.634	0.899	-8.492	9.673
			Hijaz	-0.329	4.630	0.943	-9.402	8.745
			Huzam	-0.365	4.628	0.937	-9.435	8.705
			Bayati	-0.069	4.636	0.988	-9.155	9.018
			Rast	0.078	4.559	0.986	-8.858	9.014
Beta-L	F3	Baseline	Kurd	-0.331	4.642	0.943	-9.429	8.767
			Saba	-0.563	4.631	0.903	-9.639	8.513
			Ajam	-0.642	4.633	0.890	-9.723	8.439
			Nahawand	-0.660	4.634	0.887	-9.742	8.423
			Hijaz	-1.884	4.630	0.684	-10.958	7.190
			Huzam	-2.016	4.628	0.663	-11.086	7.054
			Bayati	-1.170	4.636	0.801	-10.256	7.917
			Rast	-1.093	4.559	0.811	-10.029	7.843
Beta-H	F3	Baseline	Kurd	-0.884	4.642	0.849	-9.982	8.214
			Saba	-1.781	4.631	0.701	-10.857	7.295
			Ajam	-1.146	4.633	0.805	-10.228	7.935
			Nahawand	-1.611	4.634	0.728	-10.694	7.472
			Hijaz	-2.449	4.630	0.597	-11.523	6.625
			Huzam	-2.561	4.628	0.580	-11.631	6.509
			Bayati	-1.535	4.636	0.741	-10.622	7.551
			Rast	-1.796	4.559	0.694	-10.732	7.140
amma	F3	Baseline	Kurd	-1.104	4.642	0.812	-10.202	7.994
			Saba	-2.873	4.631	0.535	-11.949	6.203
			Ajam	-2.081	4.633	0.653	-11.163	7.000
			Nahawand	-2.224	4.634	0.631	-11.307	6.859
			Hijaz	-2.810	4.630	0.544	-11.884	6.264
			Huzam	-2.850	4.628	0.538	-11.920	6.220

				Mean Difference (I-J)	Std. Error	Sig.[b]	95% Confidence Interval for Difference[b] Lower Bound	Upper Bound
G			Bayati	-2.104	4.636	0.650	-11.190	6.983
			Rast	-2.213	4.559	0.627	-11.149	6.722

FC5

Wave	Electrode	(I)	(J)	Mean Difference (I-J)	Std. Error	Sig.[b]	95% Confidence Interval for Difference[b] Lower Bound	Upper Bound
Theta	FC5	Baseline	Kurd	1.381	4.642	0.766	-7.717	10.479
			Saba	0.559	4.631	0.904	-8.517	9.635
			Ajam	-2.501	4.633	0.589	-11.583	6.580
			Nahawand	-0.039	4.634	0.993	-9.121	9.044
			Hijaz	-4.504	4.630	0.331	-13.577	4.570
			Huzam	-0.814	4.628	0.860	-9.884	8.256
			Bayati	0.468	4.636	0.920	-8.618	9.555
			Rast	0.693	4.559	0.879	-8.243	9.628
Alpha	FC5	Baseline	Kurd	-0.124	4.642	0.979	-9.222	8.975
			Saba	-0.124	4.631	0.979	-9.200	8.952
			Ajam	-0.129	4.633	0.978	-9.210	8.953
			Nahawand	-0.522	4.634	0.910	-9.605	8.560
			Hijaz	-1.606	4.630	0.729	-10.680	7.467
			Huzam	-1.926	4.628	0.677	-10.995	7.144
			Bayati	-1.270	4.636	0.784	-10.356	7.816
			Rast	-1.173	4.559	0.797	-10.108	7.763
Beta-L	FC5	Baseline	Kurd	-0.560	4.642	0.904	-9.658	8.539
			Saba	-0.952	4.631	0.837	-10.028	8.124
			Ajam	-0.671	4.633	0.885	-9.753	8.410
			Nahawand	-1.229	4.634	0.791	-10.312	7.854
			Hijaz	-2.306	4.630	0.618	-11.380	6.768
			Huzam	-2.459	4.628	0.595	-11.529	6.611
			Bayati	-1.772	4.636	0.702	-10.858	7.315
			Rast	-1.725	4.559	0.705	-10.661	7.211
Beta-H	FC5	Baseline	Kurd	-0.861	4.642	0.853	-9.959	8.238
			Saba	-1.908	4.631	0.680	-10.984	7.168
			Ajam	-1.278	4.633	0.783	-10.359	7.803
			Nahawand	-1.890	4.634	0.683	-10.973	7.193
			Hijaz	-2.676	4.630	0.563	-11.750	6.398
			Huzam	-2.938	4.628	0.526	-12.008	6.132
			Bayati	-1.768	4.636	0.703	-10.855	7.318
			Rast	-2.019	4.559	0.658	-10.955	6.916
Gamma	FC5	Baseline	Kurd	-1.102	4.642	0.812	-10.201	7.996
			Saba	-3.055	4.631	0.509	-12.131	6.021
			Ajam	-2.254	4.633	0.627	-11.335	6.827
			Nahawand	-2.588	4.634	0.577	-11.671	6.495
			Hijaz	-3.198	4.630	0.490	-12.272	5.875
			Huzam	-3.324	4.628	0.473	-12.394	5.746
			Bayati	-2.412	4.636	0.603	-11.499	6.674

			Rast	-2.479	4.559	0.587	-11.414	6.457

T7

Wave	Electrode	(I)	(J)	Mean Difference (I-J)	Std. Error	Sig.[b]	95% Confidence Interval for Difference[b] Lower Bound	Upper Bound
**Theta**	**T7**	**Baseline**	Kurd	-8.505	4.642	0.067	-17.603	0.593
			Saba	-6.307	4.631	0.173	-15.383	2.768
			Ajam	-6.746	4.633	0.145	-15.828	2.335
			Nahawand	-7.285	4.634	0.116	-16.368	1.797
			Hijaz	-13.921*	4.630	0.003	-22.995	-4.848
			Huzam	-3.170	4.628	0.493	-12.240	5.899
			Bayati	-1.759	4.636	0.704	-10.846	7.327
			Rast	-3.695	4.559	0.418	-12.630	5.241
**Alpha**	**T7**	**Baseline**	Kurd	-2.046	4.642	0.659	-11.144	7.052
			Saba	-2.388	4.631	0.606	-11.464	6.688
			Ajam	-1.259	4.633	0.786	-10.341	7.822
			Nahawand	-1.007	4.634	0.828	-10.090	8.076
			Hijaz	-1.824	4.630	0.694	-10.898	7.249
			Huzam	-1.732	4.628	0.708	-10.802	7.338
			Bayati	-1.223	4.636	0.792	-10.309	7.864
			Rast	-1.444	4.559	0.752	-10.379	7.492
**Beta-L**	**T7**	**Baseline**	Kurd	-1.296	4.642	0.780	-10.395	7.802
			Saba	-1.950	4.631	0.674	-11.026	7.126
			Ajam	-1.104	4.633	0.812	-10.186	7.977
			Nahawand	-1.325	4.634	0.775	-10.407	7.758
			Hijaz	-2.133	4.630	0.645	-11.207	6.941
			Huzam	-2.122	4.628	0.647	-11.191	6.948
			Bayati	-1.448	4.636	0.755	-10.535	7.638
			Rast	-1.388	4.559	0.761	-10.323	7.548
**Beta-H**	**T7**	**Baseline**	Kurd	-2.836	4.642	0.541	-11.934	6.262
			Saba	-3.200	4.631	0.490	-12.276	5.876
			Ajam	-1.869	4.633	0.687	-10.950	7.213
			Nahawand	-1.985	4.634	0.668	-11.068	7.098
			Hijaz	-2.721	4.630	0.557	-11.795	6.353
			Huzam	-2.796	4.628	0.546	-11.866	6.273
			Bayati	-1.849	4.636	0.690	-10.935	7.238
			Rast	-1.892	4.559	0.678	-10.828	7.044
**amma**	**T7**	**Baseline**	Kurd	-2.553	4.642	0.582	-11.651	6.545
			Saba	-4.688	4.631	0.311	-13.764	4.388
			Ajam	-2.795	4.633	0.546	-11.877	6.286
			Nahawand	-3.052	4.634	0.510	-12.134	6.031
			Hijaz	-3.552	4.630	0.443	-12.626	5.522
			Huzam	-3.833	4.628	0.408	-12.903	5.237

Wave	Electrode	(I)	(J)	Mean Difference (I-J)	Std. Error	Sig.	Lower Bound	Upper Bound
G			Bayati	-2.782	4.636	0.548	-11.868	6.305
			Rast	-2.546	4.559	0.576	-11.482	6.389

P7

Wave	Electrode	(I)	(J)	Mean Difference (I-J)	Std. Error	Sig.[b]	95% Confidence Interval for Difference[b] Lower Bound	Upper Bound
**Theta**	**P7**	**Baseline**	Kurd	-0.443	4.642	0.924	-9.541	8.655
			Saba	-0.993	4.631	0.830	-10.068	8.083
			Ajam	-2.691	4.633	0.561	-11.773	6.390
			Nahawand	-1.556	4.634	0.737	-10.639	7.527
			Hijaz	-4.461	4.630	0.335	-13.535	4.613
			Huzam	-2.119	4.628	0.647	-11.189	6.951
			Bayati	-1.759	4.636	0.704	-10.845	7.328
			Rast	-1.064	4.559	0.815	-10.000	7.872
**Alpha**	**P7**	**Baseline**	Kurd	-1.065	4.642	0.818	-10.163	8.033
			Saba	-2.181	4.631	0.638	-11.257	6.895
			Ajam	-1.921	4.633	0.678	-11.002	7.161
			Nahawand	-2.847	4.634	0.539	-11.929	6.236
			Hijaz	-3.397	4.630	0.463	-12.471	5.676
			Huzam	-4.089	4.628	0.377	-13.159	4.981
			Bayati	-3.318	4.636	0.474	-12.404	5.769
			Rast	-2.796	4.559	0.540	-11.732	6.139
**Beta-L**	**P7**	**Baseline**	Kurd	-1.733	4.642	0.709	-10.831	7.365
			Saba	-5.261	4.631	0.256	-14.337	3.814
			Ajam	-4.031	4.633	0.384	-13.112	5.051
			Nahawand	-5.247	4.634	0.258	-14.330	3.836
			Hijaz	-6.154	4.630	0.184	-15.227	2.920
			Huzam	-7.318	4.628	0.114	-16.388	1.752
			Bayati	-5.084	4.636	0.273	-14.170	4.003
			Rast	-4.334	4.559	0.342	-13.270	4.601
**Beta-H**	**P7**	**Baseline**	Kurd	-2.519	4.642	0.587	-11.617	6.579
			Saba	-9.815*	4.631	0.034	-18.891	-0.740
			Ajam	-5.731	4.633	0.216	-14.812	3.350
			Nahawand	-6.558	4.634	0.157	-15.640	2.525
			Hijaz	-8.207	4.630	0.076	-17.281	0.867
			Huzam	-9.084*	4.628	0.050	-18.154	-0.014
			Bayati	-6.489	4.636	0.162	-15.575	2.598
			Rast	-5.625	4.559	0.217	-14.560	3.311
**mma**	**P7**	**Baseline**	Kurd	-3.382	4.642	0.466	-12.481	5.716
			Saba	-14.627*	4.631	0.002	-23.703	-5.551
			Ajam	-8.488	4.633	0.067	-17.569	0.593
			Nahawand	-9.378*	4.634	0.043	-18.461	-0.295
			Hijaz	-10.938*	4.630	0.018	-20.012	-1.864

Ga			Huzam	-12.161*	4.628	0.009	-21.231	-3.091
			Bayati	-8.726	4.636	0.060	-17.812	0.361
			Rast	-7.598	4.559	0.096	-16.533	1.338

O1

Wave	Electrode	(I)	(J)	Mean Difference (I-J)	Std. Error	Sig.[b]	95% Confidence Interval for Difference[b] Lower Bound	Upper Bound
Theta	O1	Baseline	Kurd	-0.369	4.642	0.937	-9.467	8.729
			Saba	-0.640	4.631	0.890	-9.716	8.436
			Ajam	-2.505	4.633	0.589	-11.586	6.576
			Nahawand	-0.982	4.634	0.832	-10.065	8.101
			Hijaz	-4.182	4.630	0.366	-13.256	4.892
			Huzam	-1.629	4.628	0.725	-10.699	7.441
			Bayati	-1.340	4.636	0.773	-10.426	7.747
			Rast	-0.962	4.559	0.833	-9.898	7.974
Alpha	O1	Baseline	Kurd	-0.722	4.642	0.876	-9.820	8.376
			Saba	-0.939	4.631	0.839	-10.015	8.137
			Ajam	-1.113	4.633	0.810	-10.194	7.968
			Nahawand	-1.444	4.634	0.755	-10.527	7.639
			Hijaz	-2.698	4.630	0.560	-11.772	6.376
			Huzam	-2.984	4.628	0.519	-12.054	6.086
			Bayati	-2.926	4.636	0.528	-12.013	6.160
			Rast	-2.306	4.559	0.613	-11.242	6.630
Beta-L	O1	Baseline	Kurd	-0.985	4.642	0.832	-10.083	8.113
			Saba	-1.701	4.631	0.713	-10.777	7.375
			Ajam	-1.522	4.633	0.742	-10.604	7.559
			Nahawand	-1.932	4.634	0.677	-11.015	7.151
			Hijaz	-3.271	4.630	0.480	-12.345	5.802
			Huzam	-3.479	4.628	0.452	-12.549	5.591
			Bayati	-2.685	4.636	0.562	-11.771	6.402
			Rast	-2.440	4.559	0.593	-11.375	6.496
Beta-H	O1	Baseline	Kurd	-1.168	4.642	0.801	-10.267	7.930
			Saba	-3.046	4.631	0.511	-12.122	6.030
			Ajam	-1.974	4.633	0.670	-11.055	7.108
			Nahawand	-2.405	4.634	0.604	-11.488	6.678
			Hijaz	-3.362	4.630	0.468	-12.436	5.712
			Huzam	-3.536	4.628	0.445	-12.605	5.534
			Bayati	-2.452	4.636	0.597	-11.538	6.635
			Rast	-2.585	4.559	0.571	-11.521	6.351
amma	O1	Baseline	Kurd	-1.457	4.642	0.754	-10.555	7.641
			Saba	-4.546	4.631	0.326	-13.622	4.530
			Ajam	-3.075	4.633	0.507	-12.157	6.006
			Nahawand	-3.266	4.634	0.481	-12.349	5.816
			Hijaz	-4.031	4.630	0.384	-13.104	5.043
			Huzam	-4.206	4.628	0.363	-13.276	4.864

G	Bayati	-3.101	4.636	0.504	-12.187	5.986
	Rast	-3.167	4.559	0.487	-12.103	5.768

Right

AF4

Wave	Electrode	(I)	(J)	Mean Difference (I-J)	Std. Error	Sig.[b]	95% Confidence Interval for Difference[b] Lower Bound	Upper Bound
Theta	AF4	Baseline	Kurd	-0.468	4.642	0.920	-9.566	8.630
			Saba	-0.595	4.631	0.898	-9.671	8.481
			Ajam	-2.974	4.633	0.521	-12.056	6.107
			Nahawand	-1.291	4.634	0.781	-10.374	7.791
			Hijaz	-4.820	4.630	0.298	-13.893	4.254
			Huzam	-2.218	4.628	0.632	-11.288	6.852
			Bayati	-0.836	4.636	0.857	-9.922	8.251
			Rast	-0.989	4.559	0.828	-9.925	7.946
Alpha	AF4	Baseline	Kurd	-0.542	4.642	0.907	-9.640	8.556
			Saba	-0.435	4.631	0.925	-9.511	8.641
			Ajam	-0.639	4.633	0.890	-9.720	8.442
			Nahawand	-1.031	4.634	0.824	-10.114	8.052
			Hijaz	-2.343	4.630	0.613	-11.417	6.731
			Huzam	-2.710	4.628	0.558	-11.779	6.360
			Bayati	-2.196	4.636	0.636	-11.282	6.891
			Rast	-1.832	4.559	0.688	-10.768	7.104
Beta-L	AF4	Baseline	Kurd	-0.661	4.642	0.887	-9.759	8.438
			Saba	-1.015	4.631	0.827	-10.091	8.061
			Ajam	-0.747	4.633	0.872	-9.829	8.334
			Nahawand	-1.288	4.634	0.781	-10.371	7.794
			Hijaz	-2.604	4.630	0.574	-11.677	6.470
			Huzam	-2.788	4.628	0.547	-11.858	6.282
			Bayati	-1.925	4.636	0.678	-11.012	7.161
			Rast	-1.851	4.559	0.685	-10.786	7.085
Beta-H	AF4	Baseline	Kurd	-1.054	4.642	0.820	-10.152	8.044
			Saba	-2.105	4.631	0.649	-11.181	6.971
			Ajam	-1.375	4.633	0.767	-10.456	7.706
			Nahawand	-1.994	4.634	0.667	-11.077	7.088
			Hijaz	-2.928	4.630	0.527	-12.001	6.146
			Huzam	-3.041	4.628	0.511	-12.111	6.029
			Bayati	-1.979	4.636	0.669	-11.066	7.107
			Rast	-2.233	4.559	0.624	-11.169	6.703
Gamma	AF4	Baseline	Kurd	-1.326	4.642	0.775	-10.425	7.772
			Saba	-3.318	4.631	0.474	-12.394	5.758
			Ajam	-2.428	4.633	0.600	-11.509	6.653
			Nahawand	-2.742	4.634	0.554	-11.824	6.341
			Hijaz	-3.469	4.630	0.454	-12.543	5.605
			Huzam	-3.581	4.628	0.439	-12.651	5.489
			Bayati	-2.674	4.636	0.564	-11.760	6.413

			Rast	-2.816	4.559	0.537	-11.752	6.119

F8

Wave	Electrode	(I)	(J)	Mean Difference (I-J)	Std. Error	Sig.[b]	95% Confidence Interval for Difference[b] Lower Bound	Upper Bound
Theta	F8	Baseline	Kurd	-0.922	4.642	0.843	-10.020	8.176
			Saba	-1.526	4.631	0.742	-10.601	7.550
			Ajam	-6.295	4.633	0.174	-15.376	2.787
			Nahawand	-6.513	4.634	0.160	-15.596	2.569
			Hijaz	-8.114	4.630	0.080	-17.188	0.960
			Huzam	-4.017	4.628	0.385	-13.087	5.053
			Bayati	-2.078	4.636	0.654	-11.164	7.009
			Rast	-5.519	4.559	0.226	-14.455	3.417
Alpha	F8	Baseline	Kurd	-0.895	4.642	0.847	-9.993	8.204
			Saba	-1.139	4.631	0.806	-10.215	7.937
			Ajam	-1.510	4.633	0.745	-10.591	7.572
			Nahawand	-2.040	4.634	0.660	-11.122	7.043
			Hijaz	-3.923	4.630	0.397	-12.997	5.151
			Huzam	-3.661	4.628	0.429	-12.731	5.409
			Bayati	-2.797	4.636	0.546	-11.884	6.289
			Rast	-2.715	4.559	0.551	-11.651	6.220
Beta-L	F8	Baseline	Kurd	-1.074	4.642	0.817	-10.172	8.024
			Saba	-1.767	4.631	0.703	-10.843	7.309
			Ajam	-1.470	4.633	0.751	-10.552	7.611
			Nahawand	-2.075	4.634	0.654	-11.158	7.008
			Hijaz	-3.846	4.630	0.406	-12.920	5.228
			Huzam	-3.604	4.628	0.436	-12.674	5.466
			Bayati	-2.488	4.636	0.592	-11.574	6.599
			Rast	-2.522	4.559	0.580	-11.458	6.414
Beta-H	F8	Baseline	Kurd	-1.330	4.642	0.775	-10.428	7.768
			Saba	-3.126	4.631	0.500	-12.202	5.949
			Ajam	-2.156	4.633	0.642	-11.237	6.926
			Nahawand	-2.823	4.634	0.542	-11.906	6.260
			Hijaz	-3.777	4.630	0.415	-12.850	5.297
			Huzam	-4.026	4.628	0.384	-13.096	5.044
			Bayati	-2.616	4.636	0.573	-11.703	6.470
			Rast	-2.912	4.559	0.523	-11.848	6.023
mma	F8	Baseline	Kurd	-1.742	4.642	0.708	-10.840	7.357
			Saba	-4.769	4.631	0.303	-13.845	4.307
			Ajam	-3.283	4.633	0.479	-12.364	5.798
			Nahawand	-3.824	4.634	0.409	-12.907	5.259
			Hijaz	-4.387	4.630	0.343	-13.461	4.687

			Huzam	-4.757	4.628	0.304	-13.827	4.313
Ga			Bayati	-3.306	4.636	0.476	-12.392	5.781
			Rast	-3.525	4.559	0.439	-12.461	5.411

F4

Wave	Electrode	(I)	(J)	Mean Difference (I-J)	Std. Error	Sig.[b]	95% Confidence Interval for Difference[b] Lower Bound	Upper Bound
Theta	F4	Baseline	Kurd	-0.111	4.642	0.981	-9.209	8.987
			Saba	-1.081	4.631	0.815	-10.157	7.995
			Ajam	-7.083	4.633	0.126	-16.164	1.998
			Nahawand	-1.668	4.634	0.719	-10.751	7.414
			Hijaz	-6.751	4.630	0.145	-15.825	2.322
			Huzam	-2.760	4.628	0.551	-11.830	6.310
			Bayati	-1.621	4.636	0.727	-10.707	7.466
			Rast	-1.253	4.559	0.783	-10.189	7.683
Alpha	F4	Baseline	Kurd	-0.548	4.642	0.906	-9.646	8.550
			Saba	-0.667	4.631	0.885	-9.743	8.408
			Ajam	-1.155	4.633	0.803	-10.236	7.927
			Nahawand	-1.456	4.634	0.753	-10.539	7.627
			Hijaz	-3.253	4.630	0.482	-12.327	5.821
			Huzam	-3.980	4.628	0.390	-13.050	5.090
			Bayati	-3.519	4.636	0.448	-12.606	5.567
			Rast	-2.912	4.559	0.523	-11.848	6.024
Beta-L	F4	Baseline	Kurd	-1.086	4.642	0.815	-10.184	8.012
			Saba	-1.472	4.631	0.751	-10.548	7.604
			Ajam	-1.173	4.633	0.800	-10.254	7.908
			Nahawand	-1.807	4.634	0.697	-10.890	7.276
			Hijaz	-3.751	4.630	0.418	-12.824	5.323
			Huzam	-4.101	4.628	0.376	-13.171	4.969
			Bayati	-2.996	4.636	0.518	-12.082	6.091
			Rast	-2.812	4.559	0.537	-11.748	6.123
Beta-H	F4	Baseline	Kurd	-1.340	4.642	0.773	-10.438	7.758
			Saba	-2.737	4.631	0.554	-11.813	6.338
			Ajam	-1.774	4.633	0.702	-10.855	7.307
			Nahawand	-2.562	4.634	0.580	-11.645	6.520
			Hijaz	-3.825	4.630	0.409	-12.899	5.249
			Huzam	-4.002	4.628	0.387	-13.072	5.068
			Bayati	-2.579	4.636	0.578	-11.665	6.508
			Rast	-2.963	4.559	0.516	-11.898	5.973
amma	F4	Baseline	Kurd	-1.634	4.642	0.725	-10.732	7.464
			Saba	-4.316	4.631	0.351	-13.392	4.760
			Ajam	-3.069	4.633	0.508	-12.150	6.012
			Nahawand	-3.466	4.634	0.454	-12.549	5.616
			Hijaz	-4.281	4.630	0.355	-13.355	4.793
			Huzam	-4.498	4.628	0.331	-13.568	4.572

Wave	Electrode	(I)	(J)	Mean Difference (I-J)	Std. Error	Sig.[b]	95% Confidence Interval for Difference[b] Lower Bound	Upper Bound
G			Bayati	-3.331	4.636	0.472	-12.417	5.756
			Rast	-3.523	4.559	0.440	-12.459	5.413

FC6

Wave	Electrode	(I)	(J)	Mean Difference (I-J)	Std. Error	Sig.[b]	95% Confidence Interval for Difference[b] Lower Bound	Upper Bound
Theta	FC6	Baseline	Kurd	-1.638	4.642	0.724	-10.737	7.460
			Saba	-3.145	4.631	0.497	-12.221	5.931
			Ajam	-5.411	4.633	0.243	-14.492	3.670
			Nahawand	-1.824	4.634	0.694	-10.907	7.259
			Hijaz	-6.803	4.630	0.142	-15.877	2.271
			Huzam	-2.970	4.628	0.521	-12.040	6.100
			Bayati	-1.604	4.636	0.729	-10.690	7.483
			Rast	-1.352	4.559	0.767	-10.287	7.584
Alpha	FC6	Baseline	Kurd	-1.202	4.642	0.796	-10.301	7.896
			Saba	-1.433	4.631	0.757	-10.509	7.643
			Ajam	-1.083	4.633	0.815	-10.165	7.998
			Nahawand	-1.655	4.634	0.721	-10.737	7.428
			Hijaz	-3.819	4.630	0.409	-12.893	5.255
			Huzam	-4.199	4.628	0.364	-13.269	4.871
			Bayati	-3.373	4.636	0.467	-12.459	5.714
			Rast	-2.846	4.559	0.532	-11.781	6.090
Beta-L	FC6	Baseline	Kurd	-1.400	4.642	0.763	-10.499	7.698
			Saba	-2.339	4.631	0.613	-11.415	6.737
			Ajam	-1.378	4.633	0.766	-10.459	7.704
			Nahawand	-2.142	4.634	0.644	-11.225	6.940
			Hijaz	-4.001	4.630	0.387	-13.075	5.072
			Huzam	-4.526	4.628	0.328	-13.596	4.544
			Bayati	-3.186	4.636	0.492	-12.272	5.901
			Rast	-3.041	4.559	0.505	-11.977	5.894
Beta-H	FC6	Baseline	Kurd	-1.717	4.642	0.712	-10.815	7.381
			Saba	-4.154	4.631	0.370	-13.230	4.922
			Ajam	-2.384	4.633	0.607	-11.465	6.697
			Nahawand	-3.353	4.634	0.469	-12.436	5.730
			Hijaz	-4.604	4.630	0.320	-13.678	4.470
			Huzam	-4.971	4.628	0.283	-14.040	4.099
			Bayati	-3.254	4.636	0.483	-12.341	5.832
			Rast	-3.494	4.559	0.444	-12.429	5.442
Gamma	FC6	Baseline	Kurd	-2.072	4.642	0.655	-11.170	7.026
			Saba	-6.164	4.631	0.183	-15.240	2.912
			Ajam	-3.768	4.633	0.416	-12.850	5.313
			Nahawand	-4.637	4.634	0.317	-13.720	4.446
			Hijaz	-5.265	4.630	0.255	-14.339	3.809
			Huzam	-5.824	4.628	0.208	-14.894	3.246
			Bayati	-4.189	4.636	0.366	-13.276	4.897

			Rast	-4.166	4.559	0.361	-13.101	4.770

T8

Wave	Electrode	(I)	(J)	Mean Difference (I-J)	Std. Error	Sig.[b]	95% Confidence Interval for Difference[b] Lower Bound	Upper Bound
Theta	**T8**	**Baseline**	Kurd	-0.833	4.642	0.858	-9.931	8.266
			Saba	-1.657	4.631	0.721	-10.733	7.419
			Ajam	-3.301	4.633	0.476	-12.383	5.780
			Nahawand	-2.471	4.634	0.594	-11.554	6.611
			Hijaz	-5.780	4.630	0.212	-14.853	3.294
			Huzam	-3.319	4.628	0.473	-12.389	5.751
			Bayati	-2.700	4.636	0.560	-11.787	6.386
			Rast	-2.104	4.559	0.644	-11.040	6.831
Alpha	**T8**	**Baseline**	Kurd	-1.237	4.642	0.790	-10.335	7.861
			Saba	-2.393	4.631	0.605	-11.468	6.683
			Ajam	-2.058	4.633	0.657	-11.139	7.024
			Nahawand	-3.016	4.634	0.515	-12.098	6.067
			Hijaz	-4.388	4.630	0.343	-13.462	4.686
			Huzam	-5.341	4.628	0.248	-14.411	3.729
			Bayati	-4.452	4.636	0.337	-13.539	4.634
			Rast	-3.733	4.559	0.413	-12.668	5.203
Beta-L	**T8**	**Baseline**	Kurd	-2.446	4.642	0.598	-11.544	6.653
			Saba	-5.759	4.631	0.214	-14.835	3.317
			Ajam	-3.918	4.633	0.398	-12.999	5.163
			Nahawand	-5.789	4.634	0.212	-14.872	3.293
			Hijaz	-6.783	4.630	0.143	-15.857	2.291
			Huzam	-8.245	4.628	0.075	-17.315	0.825
			Bayati	-6.284	4.636	0.175	-15.371	2.802
			Rast	-5.093	4.559	0.264	-14.029	3.842
Beta-H	**T8**	**Baseline**	Kurd	-3.737	4.642	0.421	-12.836	5.361
			Saba	-15.467*	4.631	0.001	-24.543	-6.391
			Ajam	-7.904	4.633	0.088	-16.986	1.177
			Nahawand	-10.566*	4.634	0.023	-19.649	-1.483
			Hijaz	-11.385*	4.630	0.014	-20.459	-2.311
			Huzam	-13.460*	4.628	0.004	-22.530	-4.390
			Bayati	-10.159*	4.636	0.028	-19.245	-1.073
			Rast	-8.060	4.559	0.077	-16.995	0.876
amma	**T8**	**Baseline**	Kurd	-5.791	4.642	0.212	-14.889	3.307
			Saba	-24.644*	4.631	0.000	-33.720	-15.568
			Ajam	-11.948*	4.633	0.010	-21.030	-2.867
			Nahawand	-16.524*	4.634	0.000	-25.607	-7.441
			Hijaz	-16.856*	4.630	0.000	-25.930	-7.782
			Huzam	-20.145*	4.628	0.000	-29.215	-11.075

G			Bayati	-14.697*	4.636	0.002	-23.784	-5.611
			Rast	-11.766*	4.559	0.010	-20.701	-2.830

P8

Wave	Electrode	(I)	(J)	Mean Difference (I-J)	Std. Error	Sig.[b]	95% Confidence Interval for Difference[b] Lower Bound	Upper Bound
Theta	**P8**	**Baseline**	Kurd	-2.812	4.642	0.545	-11.911	6.286
			Saba	-1.958	4.631	0.672	-11.034	7.118
			Ajam	-3.951	4.633	0.394	-13.032	5.130
			Nahawand	-2.059	4.634	0.657	-11.142	7.023
			Hijaz	-5.786	4.630	0.211	-14.860	3.288
			Huzam	-3.308	4.628	0.475	-12.378	5.762
			Bayati	-2.866	4.636	0.536	-11.952	6.221
			Rast	-2.306	4.559	0.613	-11.242	6.630
Alpha	**P8**	**Baseline**	Kurd	-2.159	4.642	0.642	-11.257	6.940
			Saba	-1.927	4.631	0.677	-11.003	7.148
			Ajam	-2.192	4.633	0.636	-11.273	6.890
			Nahawand	-2.423	4.634	0.601	-11.506	6.660
			Hijaz	-4.358	4.630	0.347	-13.432	4.716
			Huzam	-5.244	4.628	0.257	-14.314	3.825
			Bayati	-4.808	4.636	0.300	-13.894	4.279
			Rast	-3.944	4.559	0.387	-12.879	4.992
Beta-L	**P8**	**Baseline**	Kurd	-2.544	4.642	0.584	-11.642	6.555
			Saba	-2.693	4.631	0.561	-11.769	6.383
			Ajam	-2.797	4.633	0.546	-11.878	6.285
			Nahawand	-2.815	4.634	0.544	-11.898	6.267
			Hijaz	-4.846	4.630	0.295	-13.920	4.228
			Huzam	-5.402	4.628	0.243	-14.471	3.668
			Bayati	-4.449	4.636	0.337	-13.536	4.637
			Rast	-3.866	4.559	0.396	-12.802	5.070
Beta-H	**P8**	**Baseline**	Kurd	-2.229	4.642	0.631	-11.327	6.869
			Saba	-4.129	4.631	0.373	-13.205	4.947
			Ajam	-3.185	4.633	0.492	-12.266	5.896
			Nahawand	-3.581	4.634	0.440	-12.663	5.502
			Hijaz	-4.837	4.630	0.296	-13.911	4.237
			Huzam	-5.155	4.628	0.265	-14.225	3.915
			Bayati	-3.916	4.636	0.398	-13.003	5.170
			Rast	-4.112	4.559	0.367	-13.047	4.824
mma	**P8**	**Baseline**	Kurd	-2.215	4.642	0.633	-11.313	6.883
			Saba	-5.642	4.631	0.223	-14.718	3.434
			Ajam	-4.137	4.633	0.372	-13.218	4.944
			Nahawand	-4.209	4.634	0.364	-13.292	4.873
			Hijaz	-5.265	4.630	0.255	-14.339	3.809

Wave	Electrode	(I)	(J)	Mean Difference (I-J)	Std. Error	Sig.[b]	95% Confidence Interval for Difference[b] Lower Bound	Upper Bound
Ga			Huzam	-5.739	4.628	0.215	-14.809	3.331
			Bayati	-4.332	4.636	0.350	-13.419	4.754
			Rast	-4.464	4.559	0.327	-13.400	4.471

O2

Wave	Electrode	(I)	(J)	Mean Difference (I-J)	Std. Error	Sig.[b]	95% Confidence Interval for Difference[b] Lower Bound	Upper Bound
Theta	O2	Baseline	Kurd	-0.680	4.642	0.883	-9.779	8.418
			Saba	-0.712	4.631	0.878	-9.788	8.364
			Ajam	-2.371	4.633	0.609	-11.452	6.710
			Nahawand	-1.113	4.634	0.810	-10.196	7.970
			Hijaz	-4.171	4.630	0.368	-13.245	4.902
			Huzam	-1.903	4.628	0.681	-10.973	7.167
			Bayati	-1.508	4.636	0.745	-10.594	7.579
			Rast	-1.019	4.559	0.823	-9.955	7.916
Alpha	O2	Baseline	Kurd	-0.752	4.642	0.871	-9.850	8.346
			Saba	-0.726	4.631	0.875	-9.802	8.350
			Ajam	-1.171	4.633	0.800	-10.253	7.910
			Nahawand	-1.279	4.634	0.782	-10.362	7.803
			Hijaz	-2.756	4.630	0.552	-11.830	6.318
			Huzam	-3.269	4.628	0.480	-12.339	5.801
			Bayati	-3.354	4.636	0.469	-12.441	5.732
			Rast	-2.579	4.559	0.572	-11.514	6.357
Beta-L	O2	Baseline	Kurd	-1.254	4.642	0.787	-10.352	7.845
			Saba	-1.349	4.631	0.771	-10.425	7.726
			Ajam	-1.423	4.633	0.759	-10.504	7.659
			Nahawand	-1.500	4.634	0.746	-10.583	7.582
			Hijaz	-3.052	4.630	0.510	-12.126	6.022
			Huzam	-3.270	4.628	0.480	-12.340	5.800
			Bayati	-2.661	4.636	0.566	-11.748	6.425
			Rast	-2.460	4.559	0.590	-11.395	6.476
Beta-H	O2	Baseline	Kurd	-1.141	4.642	0.806	-10.239	7.957
			Saba	-1.798	4.631	0.698	-10.874	7.278
			Ajam	-1.424	4.633	0.759	-10.506	7.657
			Nahawand	-1.734	4.634	0.708	-10.817	7.349
			Hijaz	-2.648	4.630	0.567	-11.722	6.426
			Huzam	-2.699	4.628	0.560	-11.769	6.371
			Bayati	-1.925	4.636	0.678	-11.011	7.162
			Rast	-2.230	4.559	0.625	-11.166	6.705
amma	O2	Baseline	Kurd	-1.111	4.642	0.811	-10.209	7.987
			Saba	-2.483	4.631	0.592	-11.559	6.593
			Ajam	-1.958	4.633	0.673	-11.040	7.123
			Nahawand	-2.067	4.634	0.656	-11.150	7.016
			Hijaz	-2.627	4.630	0.570	-11.701	6.447
			Huzam	-2.769	4.628	0.550	-11.839	6.301

G	Bayati	-2.131	4.636	0.646	-11.217	6.956
	Rast	-2.308	4.559	0.613	-11.243	6.628

Table T1: Mean power spectra of theta, alpha, beta-low, beta-high and gamma at F7 (frontal left): a pairwise comparisons between baseline and each *maqam*.

	F7							
Wave	Electrode	(I)	(J)	Mean Difference (I-J)	Std. Error	Sig.[b]	95% Confidence Interval for Difference[b]	
							Lower Bound	Upper Bound
**Theta**	**F7**	**Baseline**	Kurd	-42.385*	4.642	0.000	-51.483	-33.287
			Saba	-7.524	4.631	0.104	-16.600	1.552
			Ajam	-15.234*	4.633	0.001	-24.316	-6.153
			Nahawand	-48.545*	4.634	0.000	-57.628	-39.462
			Hijaz	-108.154*	4.630	0.000	-117.228	-99.080
			Huzam	-27.830*	4.628	0.000	-36.900	-18.760
			Bayati	-55.474*	4.636	0.000	-64.561	-46.388
			Rast	-85.306*	4.559	0.000	-94.242	-76.371
**Alpha**	**F7**	**Baseline**	Kurd	-5.114	4.642	0.271	-14.212	3.984
			Saba	0.859	4.631	0.853	-8.217	9.935
			Ajam	1.138	4.633	0.806	-7.943	10.219
			Nahawand	-8.837	4.634	0.057	-17.919	0.246
			Hijaz	-7.711	4.630	0.096	-16.784	1.363
			Huzam	-5.657	4.628	0.222	-14.727	3.413
			Bayati	-9.568*	4.636	0.039	-18.655	-0.482
			Rast	-8.826	4.559	0.053	-17.761	0.110
**Beta-L**	**F7**	**Baseline**	Kurd	-1.476	4.642	0.751	-10.574	7.622
			Saba	-0.390	4.631	0.933	-9.466	8.686
			Ajam	0.007	4.633	0.999	-9.074	9.089
			Nahawand	-2.584	4.634	0.577	-11.667	6.499
			Hijaz	-3.761	4.630	0.417	-12.834	5.313
			Huzam	-2.809	4.628	0.544	-11.879	6.261
			Bayati	-2.689	4.636	0.562	-11.776	6.397
			Rast	-2.674	4.559	0.557	-11.610	6.261
**Beta-H**	**F7**	**Baseline**	Kurd	-0.888	4.642	0.848	-9.986	8.210
			Saba	-1.766	4.631	0.703	-10.842	7.310
			Ajam	-1.294	4.633	0.780	-10.376	7.787
			Nahawand	-2.355	4.634	0.611	-11.438	6.728
			Hijaz	-3.524	4.630	0.447	-12.598	5.550
			Huzam	-3.335	4.628	0.471	-12.405	5.735
			Bayati	-1.800	4.636	0.698	-10.887	7.286
			Rast	-2.332	4.559	0.609	-11.268	6.604
**Gamma**	**F7**	**Baseline**	Kurd	-1.111	4.642	0.811	-10.209	7.987
			Saba	-3.190	4.631	0.491	-12.266	5.885
			Ajam	-2.491	4.633	0.591	-11.573	6.590
			Nahawand	-2.935	4.634	0.526	-12.018	6.148
			Hijaz	-3.871	4.630	0.403	-12.945	5.202
			Huzam	-4.172	4.628	0.367	-13.242	4.898
			Bayati	-2.660	4.636	0.566	-11.747	6.426
			Rast	-2.980	4.559	0.513	-11.916	5.955

Table T2: Mean power spectra of theta, alpha, beta-low, beta-high and gamma at F3 (frontal left): a pairwise comparisons between baseline and each *maqam*.

F3							95% Confidence Interval for Difference[b]	
Wave	Electrode	(I)	(J)	Mean Difference (I-J)	Std. Error	Sig.[b]	Lower Bound	Upper Bound
Theta	F3	Baseline	Kurd	0.187	4.642	0.968	-8.911	9.285
			Saba	0.309	4.631	0.947	-8.767	9.385
			Ajam	-21.235*	4.633	0.000	-30.317	-12.154
			Nahawand	5.691	4.634	0.219	-3.391	14.774
			Hijaz	2.581	4.630	0.577	-6.493	11.655
			Huzam	6.455	4.628	0.163	-2.615	15.525
			Bayati	7.097	4.636	0.126	-1.989	16.184
			Rast	6.782	4.559	0.137	-2.153	15.718
Alpha	F3	Baseline	Kurd	0.329	4.642	0.944	-8.769	9.427
			Saba	0.105	4.631	0.982	-8.971	9.180
			Ajam	-3.241	4.633	0.484	-12.322	5.841
			Nahawand	0.591	4.634	0.899	-8.492	9.673
			Hijaz	-0.329	4.630	0.943	-9.402	8.745
			Huzam	-0.365	4.628	0.937	-9.435	8.705
			Bayati	-0.069	4.636	0.988	-9.155	9.018
			Rast	0.078	4.559	0.986	-8.858	9.014
Beta-L	F3	Baseline	Kurd	-0.331	4.642	0.943	-9.429	8.767
			Saba	-0.563	4.631	0.903	-9.639	8.513
			Ajam	-0.642	4.633	0.890	-9.723	8.439
			Nahawand	-0.660	4.634	0.887	-9.742	8.423
			Hijaz	-1.884	4.630	0.684	-10.958	7.190
			Huzam	-2.016	4.628	0.663	-11.086	7.054
			Bayati	-1.170	4.636	0.801	-10.256	7.917
			Rast	-1.093	4.559	0.811	-10.029	7.843
Beta-H	F3	Baseline	Kurd	-0.884	4.642	0.849	-9.982	8.214
			Saba	-1.781	4.631	0.701	-10.857	7.295
			Ajam	-1.146	4.633	0.805	-10.228	7.935
			Nahawand	-1.611	4.634	0.728	-10.694	7.472
			Hijaz	-2.449	4.630	0.597	-11.523	6.625
			Huzam	-2.561	4.628	0.580	-11.631	6.509
			Bayati	-1.535	4.636	0.741	-10.622	7.551
			Rast	-1.796	4.559	0.694	-10.732	7.140
Gamma	F3	Baseline	Kurd	-1.104	4.642	0.812	-10.202	7.994
			Saba	-2.873	4.631	0.535	-11.949	6.203
			Ajam	-2.081	4.633	0.653	-11.163	7.000
			Nahawand	-2.224	4.634	0.631	-11.307	6.859
			Hijaz	-2.810	4.630	0.544	-11.884	6.264

			Mean Difference	Std. Error	Sig.	Lower Bound	Upper Bound
		Huzam	-2.850	4.628	0.538	-11.920	6.220
		Bayati	-2.104	4.636	0.650	-11.190	6.983
		Rast	-2.213	4.559	0.627	-11.149	6.722

Table T3: Mean power spectra of theta, alpha, beta-low, beta-high and gamma at T7 (temporal left): a pairwise comparisons between baseline and each *maqam*.

T7								
Band	Electrode	(I)	(J)	Mean Difference (I-J)	Std. Error	Sig.[b]	95% Confidence Interval for Difference[b]	
							Lower Bound	Upper Bound
Theta	**T7**	**Baseline**	Kurd	-8.505	4.642	0.067	-17.603	0.593
			Saba	-6.307	4.631	0.173	-15.383	2.768
			Ajam	-6.746	4.633	0.145	-15.828	2.335
			Nahawand	-7.285	4.634	0.116	-16.368	1.797
			Hijaz	-13.921*	4.630	0.003	-22.995	-4.848
			Huzam	-3.170	4.628	0.493	-12.240	5.899
			Bayati	-1.759	4.636	0.704	-10.846	7.327
			Rast	-3.695	4.559	0.418	-12.630	5.241
Alpha	**T7**	**Baseline**	Kurd	-2.046	4.642	0.659	-11.144	7.052
			Saba	-2.388	4.631	0.606	-11.464	6.688
			Ajam	-1.259	4.633	0.786	-10.341	7.822
			Nahawand	-1.007	4.634	0.828	-10.090	8.076
			Hijaz	-1.824	4.630	0.694	-10.898	7.249
			Huzam	-1.732	4.628	0.708	-10.802	7.338
			Bayati	-1.223	4.636	0.792	-10.309	7.864
			Rast	-1.444	4.559	0.752	-10.379	7.492
Beta-L	**T7**	**Baseline**	Kurd	-1.296	4.642	0.780	-10.395	7.802
			Saba	-1.950	4.631	0.674	-11.026	7.126
			Ajam	-1.104	4.633	0.812	-10.186	7.977
			Nahawand	-1.325	4.634	0.775	-10.407	7.758
			Hijaz	-2.133	4.630	0.645	-11.207	6.941
			Huzam	-2.122	4.628	0.647	-11.191	6.948
			Bayati	-1.448	4.636	0.755	-10.535	7.638
			Rast	-1.388	4.559	0.761	-10.323	7.548
Beta-H	**T7**	**Baseline**	Kurd	-2.836	4.642	0.541	-11.934	6.262
			Saba	-3.200	4.631	0.490	-12.276	5.876
			Ajam	-1.869	4.633	0.687	-10.950	7.213
			Nahawand	-1.985	4.634	0.668	-11.068	7.098
			Hijaz	-2.721	4.630	0.557	-11.795	6.353
			Huzam	-2.796	4.628	0.546	-11.866	6.273
			Bayati	-1.849	4.636	0.690	-10.935	7.238
			Rast	-1.892	4.559	0.678	-10.828	7.044
Gamma	**T7**	**Baseline**	Kurd	-2.553	4.642	0.582	-11.651	6.545
			Saba	-4.688	4.631	0.311	-13.764	4.388

			Ajam	-2.795	4.633	0.546	-11.877	6.286
			Nahawand	-3.052	4.634	0.510	-12.134	6.031
			Hijaz	-3.552	4.630	0.443	-12.626	5.522
			Huzam	-3.833	4.628	0.408	-12.903	5.237
			Bayati	-2.782	4.636	0.548	-11.868	6.305
			Rast	-2.546	4.559	0.576	-11.482	6.389

Table T4: Mean power spectra of theta, alpha, beta-low, beta-high and gamma at P7 (parietal left): a pairwise comparisons between baseline and each *maqam*.

P7								
Wave	Electrode	(I)	(J)	Mean Difference (I-J)	Std. Error	Sig.[b]	95% Confidence Interval for Difference[b]	
							Lower Bound	Upper Bound
**Theta**	**P7**	**Baseline**	Kurd	-0.443	4.642	0.924	-9.541	8.655
			Saba	-0.993	4.631	0.830	-10.068	8.083
			Ajam	-2.691	4.633	0.561	-11.773	6.390
			Nahawand	-1.556	4.634	0.737	-10.639	7.527
			Hijaz	-4.461	4.630	0.335	-13.535	4.613
			Huzam	-2.119	4.628	0.647	-11.189	6.951
			Bayati	-1.759	4.636	0.704	-10.845	7.328
			Rast	-1.064	4.559	0.815	-10.000	7.872
**Alpha**	**P7**	**Baseline**	Kurd	-1.065	4.642	0.818	-10.163	8.033
			Saba	-2.181	4.631	0.638	-11.257	6.895
			Ajam	-1.921	4.633	0.678	-11.002	7.161
			Nahawand	-2.847	4.634	0.539	-11.929	6.236
			Hijaz	-3.397	4.630	0.463	-12.471	5.676
			Huzam	-4.089	4.628	0.377	-13.159	4.981
			Bayati	-3.318	4.636	0.474	-12.404	5.769
			Rast	-2.796	4.559	0.540	-11.732	6.139
**Beta-L**	**P7**	**Baseline**	Kurd	-1.733	4.642	0.709	-10.831	7.365
			Saba	-5.261	4.631	0.256	-14.337	3.814
			Ajam	-4.031	4.633	0.384	-13.112	5.051
			Nahawand	-5.247	4.634	0.258	-14.330	3.836
			Hijaz	-6.154	4.630	0.184	-15.227	2.920
			Huzam	-7.318	4.628	0.114	-16.388	1.752
			Bayati	-5.084	4.636	0.273	-14.170	4.003
			Rast	-4.334	4.559	0.342	-13.270	4.601
**Beta-H**	**P7**	**Baseline**	Kurd	-2.519	4.642	0.587	-11.617	6.579
			Saba	-9.815*	4.631	0.034	-18.891	-0.740
			Ajam	-5.731	4.633	0.216	-14.812	3.350
			Nahawand	-6.558	4.634	0.157	-15.640	2.525
			Hijaz	-8.207	4.630	0.076	-17.281	0.867
			Huzam	-9.084*	4.628	0.050	-18.154	-0.014
			Bayati	-6.489	4.636	0.162	-15.575	2.598
			Rast	-5.625	4.559	0.217	-14.560	3.311

Gamma	**P7**	**Baseline**	Kurd	-3.382	4.642	0.466	-12.481	5.716
			Saba	-14.627*	4.631	0.002	-23.703	-5.551
			Ajam	-8.488	4.633	0.067	-17.569	0.593
			Nahawand	-9.378*	4.634	0.043	-18.461	-0.295
			Hijaz	-10.938*	4.630	0.018	-20.012	-1.864
			Huzam	-12.161*	4.628	0.009	-21.231	-3.091
			Bayati	-8.726	4.636	0.060	-17.812	0.361
			Rast	-7.598	4.559	0.096	-16.533	1.338

Table T5: Mean power spectra of theta, alpha, beta-low, beta-high and gamma at T8 (temporal right): a pairwise comparisons between baseline and each *maqam*.

T8								
Wave	Electrode	(I)	(J)	Mean Difference (I-J)	Std. Error	Sig.[b]	95% Confidence Interval for Difference[b]	
							Lower Bound	Upper Bound
Theta	**T8**	**Baseline**	Kurd	-0.833	4.642	0.858	-9.931	8.266
			Saba	-1.657	4.631	0.721	-10.733	7.419
			Ajam	-3.301	4.633	0.476	-12.383	5.780
			Nahawand	-2.471	4.634	0.594	-11.554	6.611
			Hijaz	-5.780	4.630	0.212	-14.853	3.294
			Huzam	-3.319	4.628	0.473	-12.389	5.751
			Bayati	-2.700	4.636	0.560	-11.787	6.386
			Rast	-2.104	4.559	0.644	-11.040	6.831
Alpha	**T8**	**Baseline**	Kurd	-1.237	4.642	0.790	-10.335	7.861
			Saba	-2.393	4.631	0.605	-11.468	6.683
			Ajam	-2.058	4.633	0.657	-11.139	7.024
			Nahawand	-3.016	4.634	0.515	-12.098	6.067
			Hijaz	-4.388	4.630	0.343	-13.462	4.686
			Huzam	-5.341	4.628	0.248	-14.411	3.729
			Bayati	-4.452	4.636	0.337	-13.539	4.634
			Rast	-3.733	4.559	0.413	-12.668	5.203
Beta-L	**T8**	**Baseline**	Kurd	-2.446	4.642	0.598	-11.544	6.653
			Saba	-5.759	4.631	0.214	-14.835	3.317
			Ajam	-3.918	4.633	0.398	-12.999	5.163
			Nahawand	-5.789	4.634	0.212	-14.872	3.293
			Hijaz	-6.783	4.630	0.143	-15.857	2.291
			Huzam	-8.245	4.628	0.075	-17.315	0.825
			Bayati	-6.284	4.636	0.175	-15.371	2.802
			Rast	-5.093	4.559	0.264	-14.029	3.842

**Beta-H**	**T8**	**Baseline**	Kurd	-3.737	4.642	0.421	-12.836	5.361
			Saba	-15.467*	4.631	0.001	-24.543	-6.391
			Ajam	-7.904	4.633	0.088	-16.986	1.177
			Nahawand	-10.566*	4.634	0.023	-19.649	-1.483
			Hijaz	-11.385*	4.630	0.014	-20.459	-2.311
			Huzam	-13.460*	4.628	0.004	-22.530	-4.390
			Bayati	-10.159*	4.636	0.028	-19.245	-1.073
			Rast	-8.060	4.559	0.077	-16.995	0.876
**Gamma**	**T8**	**Baseline**	Kurd	-5.791	4.642	0.212	-14.889	3.307
			Saba	-24.644*	4.631	0.000	-33.720	-15.568
			Ajam	-11.948*	4.633	0.010	-21.030	-2.867
			Nahawand	-16.524*	4.634	0.000	-25.607	-7.441
			Hijaz	-16.856*	4.630	0.000	-25.930	-7.782
			Huzam	-20.145*	4.628	0.000	-29.215	-11.075
			Bayati	-14.697*	4.636	0.002	-23.784	-5.611
			Rast	-11.766*	4.559	0.010	-20.701	-2.830
