# Supplementary figures and images for: EEG Correlates of Middle Eastern Music Improvisations on the Ney Instrument
Source: Front Psychol. 2021 Oct 4;12:701761. doi: 10.3389/fpsyg.2021.701761 (PMC8520950; doi:10.3389/fpsyg.2021.701761)

# PREFRONTAL

# FRONTAL

# FRONTO-CENTRAL

# TEMPORAL

# PARIETAL

# OCCIPITAL

## LEFT

## RIGHT

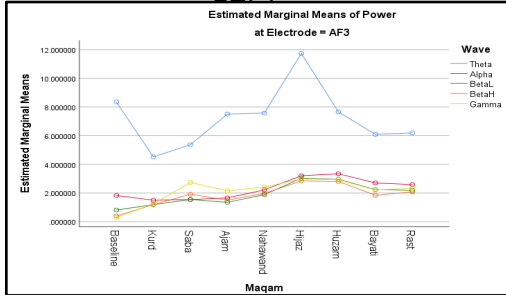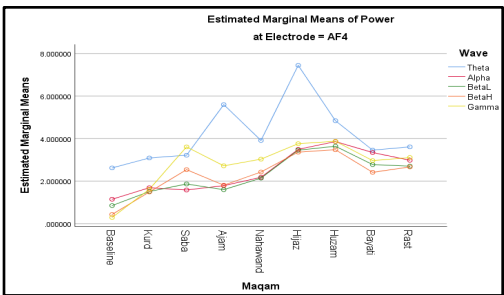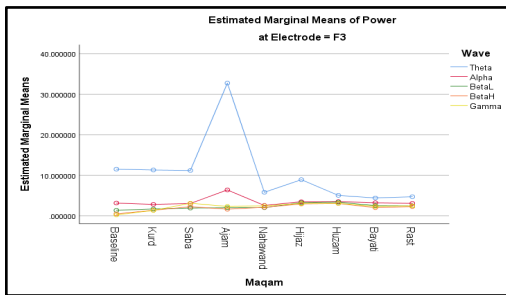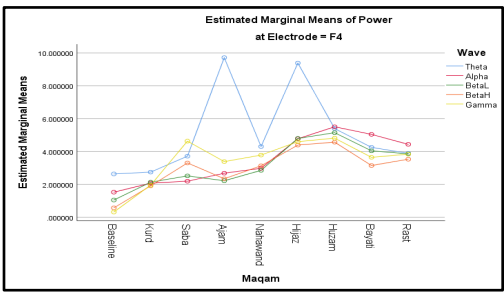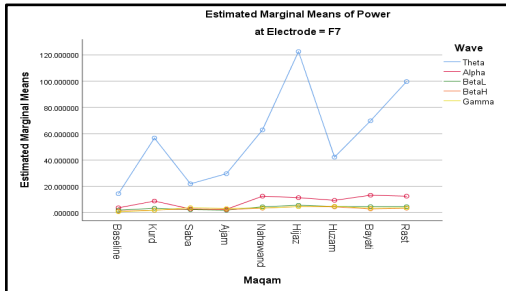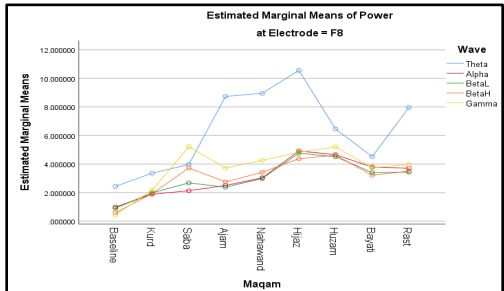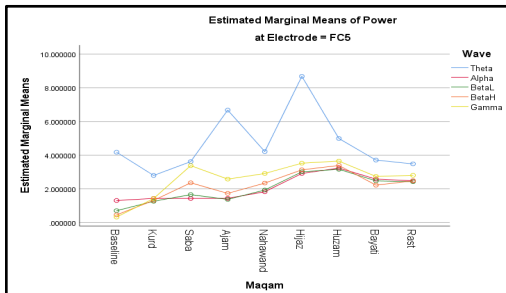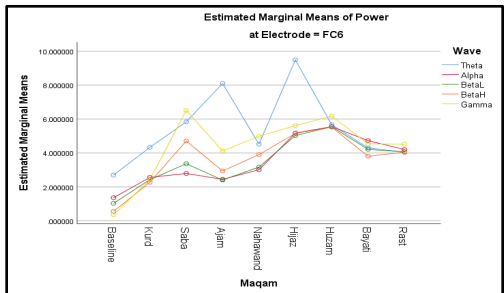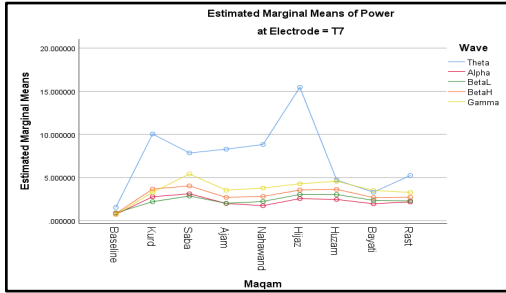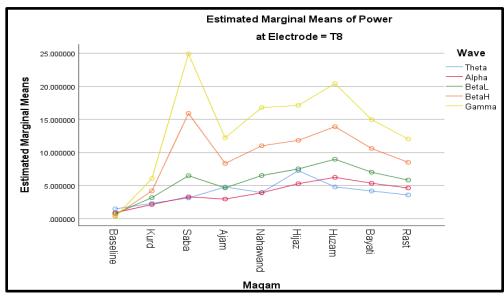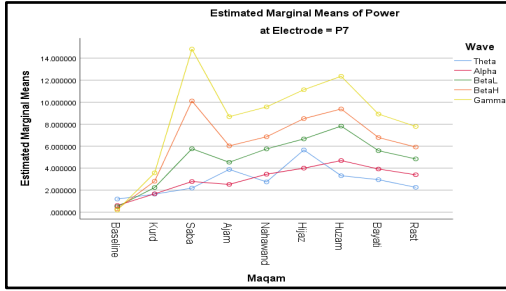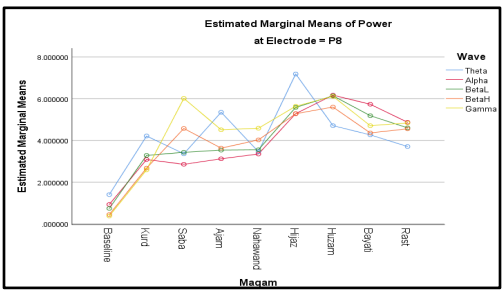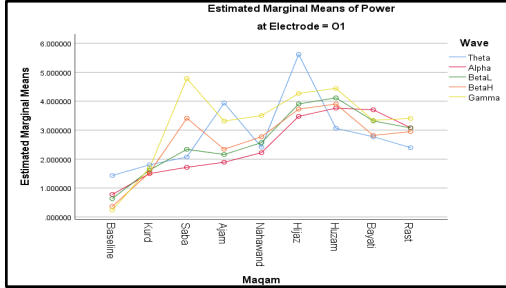

Supplement: Supplementary File 3 — Detail of the power spectra of theta, alpha, beta-low, beta-high, and gamma bands at the 3 different tempos tested, at the 14 electrode sites and for all maqams. [file Data_Sheet_3.PDF]

60 bpm

100 bpm

120 bpm

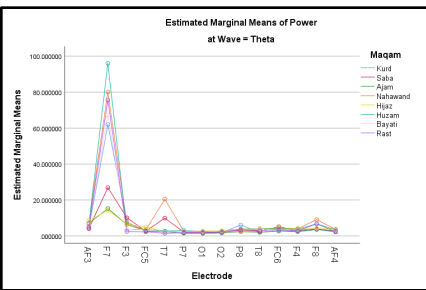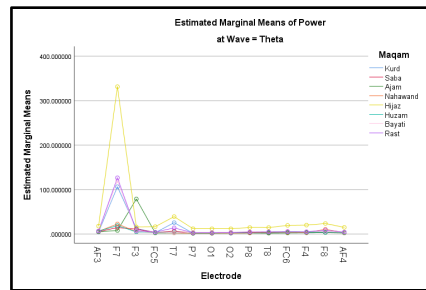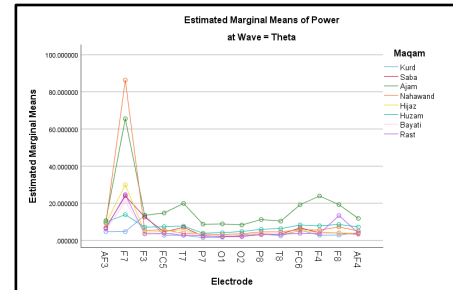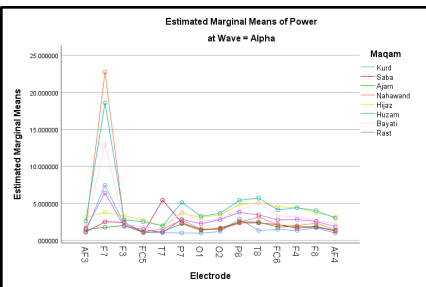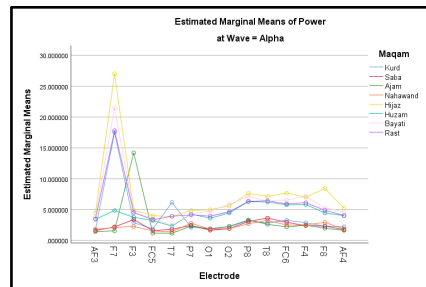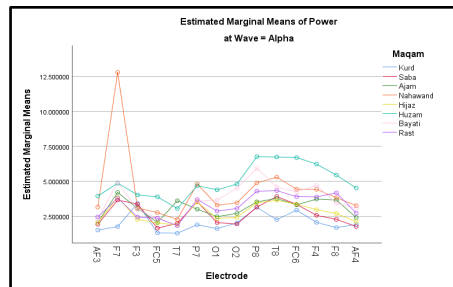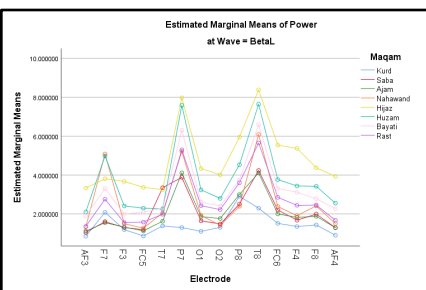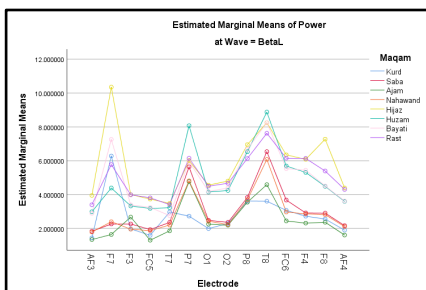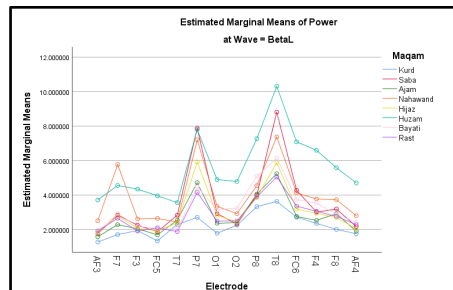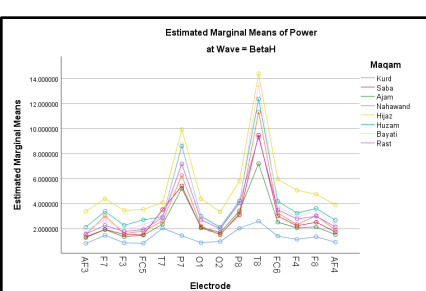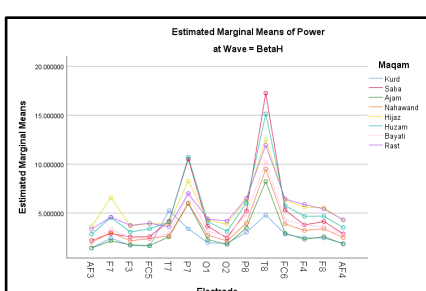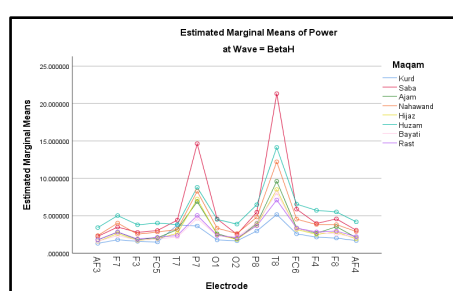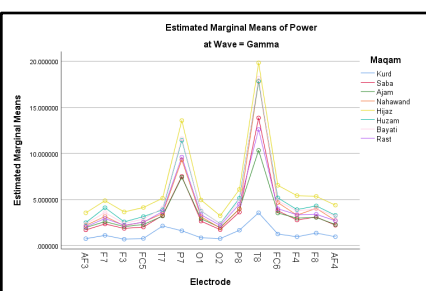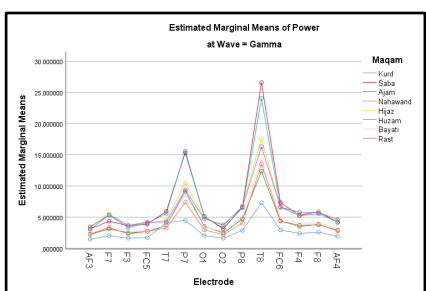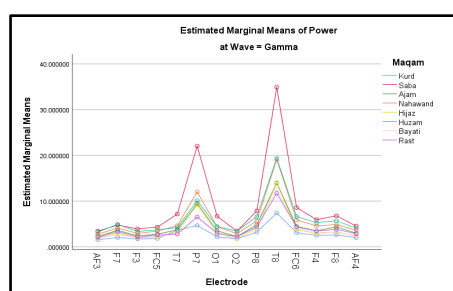

Supplement: Supplementary File 4 — Mean power spectra of theta, alpha, beta-low, beta-high, and gamma bands at the 14 electrodes organized by maqam. [file Data_Sheet_4.PDF]

Kurd

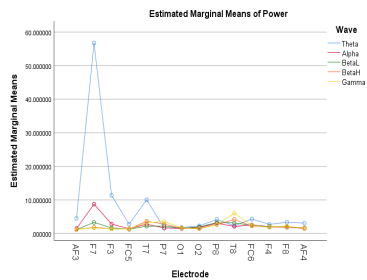

Saba

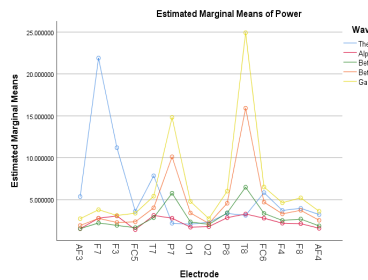

Ajam

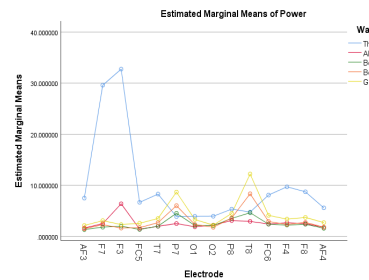

Nahawand

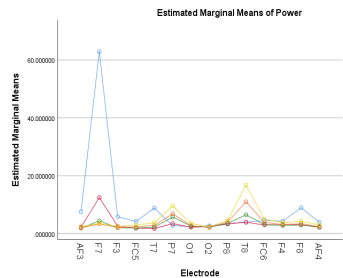

Hijaz

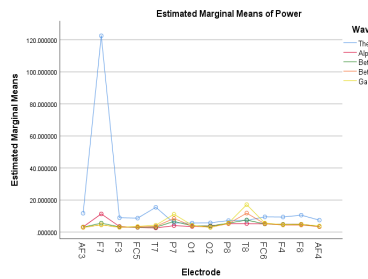

Huzam

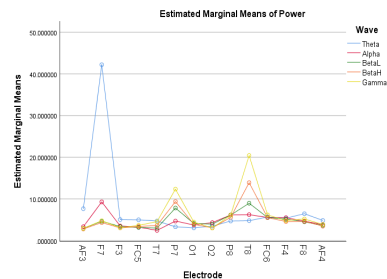

Bayati

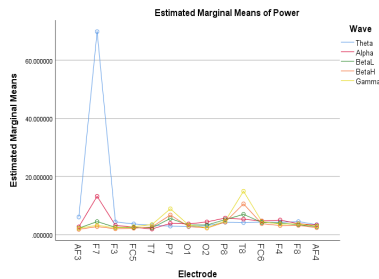

Rast

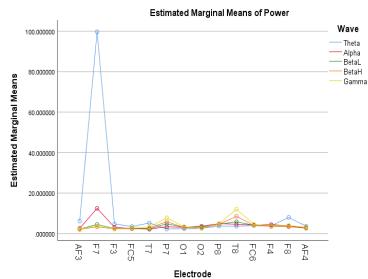

Supplement: Supplementary File 5 — Detailed output of principal component analysis. [file Data_Sheet_5.PDF]
