# Supplementary material for: EEG Correlates of Middle Eastern Music Improvisations on the Ney Instrument
Source: Front Psychol. 2021 Oct 4;12:701761. doi: 10.3389/fpsyg.2021.701761 (PMC8520950; doi:10.3389/fpsyg.2021.701761)
Supplement: Supplementary Audios — Improvisations played are available in Supplementary Files. [file Table_1.DOC]

**Factor Analysis**

| **KMO and Bartlett's Test** | | |
| --- | --- | --- |
| Kaiser-Meyer-Olkin Measure of Sampling Adequacy. | | .505 |
| Bartlett's Test of Sphericity | Approx. Chi-Square | 115.452 |
|  | df | 28 |
|  | Sig. | .000 |

| **Total Variance Explained** | | | | | | |
| --- | --- | --- | --- | --- | --- | --- |
| Component | Initial Eigenvalues | | | Extraction Sums of Squared Loadings | | |
|  | Total | % of Variance | Cumulative % | Total | % of Variance | Cumulative % |
| 1 | 1.032 | 12.900 | 12.900 | 1.032 | 12.900 | 12.900 |
| 2 | 1.020 | 12.749 | 25.648 | 1.020 | 12.749 | 25.648 |
| 3 | 1.005 | 12.565 | 38.214 | 1.005 | 12.565 | 38.214 |
| 4 | 1.000 | 12.505 | 50.719 | 1.000 | 12.505 | 50.719 |
| 5 | .991 | 12.392 | 63.111 |  |  |  |
| 6 | .988 | 12.345 | 75.456 |  |  |  |
| 7 | .984 | 12.305 | 87.761 |  |  |  |
| 8 | .979 | 12.239 | 100.000 |  |  |  |

| Extraction Method: Principal Component Analysis. |
| --- |


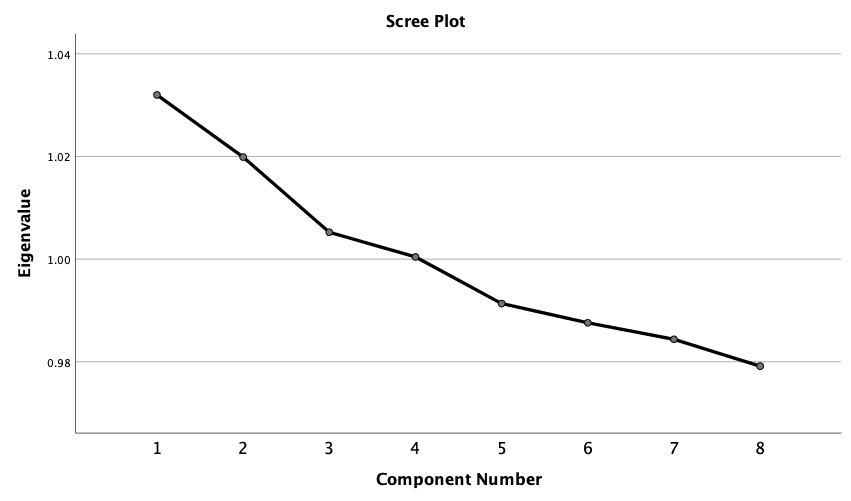


| **Communalities** | | |
| --- | --- | --- |
|  | Initial | Extraction |
| Kurd | 1.000 | .962 |
| Saba | 1.000 | .366 |
| Ajam | 1.000 | .605 |
| Nawahand | 1.000 | .511 |
| Hijaz | 1.000 | .340 |
| Huzam | 1.000 | .481 |
| Bayati | 1.000 | .448 |
| Rast | 1.000 | .344 |

| Extraction Method: Principal Component Analysis. |
| --- |

| **Component Matrix^a^** | | | | |
| --- | --- | --- | --- | --- |
|  | Component | | | |
|  | 1 | 2 | 3 | 4 |
| Saba | .591 |  |  |  |
| Rast | .568 |  |  |  |
| Hijaz | .561 |  |  |  |
| Nawahand |  | .619 | -.354 |  |
| Huzam |  | .616 |  |  |
| Ajam |  |  | .749 |  |
| Bayati |  | .420 | .505 |  |
| Kurd |  |  |  | .975 |

| Extraction Method: Principal Component Analysis.^a^ |
| --- |
| a. 4 components extracted. |

| **Pattern Matrix^a^** | | | | |
| --- | --- | --- | --- | --- |
|  | Component | | | |
|  | 1 | 2 | 3 | 4 |
| Saba | .587 |  |  |  |
| Rast | .579 |  |  |  |
| Hijaz | .579 |  |  |  |
| Nawahand |  | .708 |  |  |
| Huzam |  | .683 |  |  |
| Ajam |  |  | .764 |  |
| Bayati |  |  | .644 |  |
| Kurd |  |  |  | .981 |

| Extraction Method: Principal Component Analysis.  Rotation Method: Oblimin with Kaiser Normalization.^a^ |
| --- |
| a. Rotation converged in 4 iterations. |

| **Component Correlation Matrix** | | | | |
| --- | --- | --- | --- | --- |
| Component | 1 | 2 | 3 | 4 |
| 1 | 1.000 | .007 | .011 | -.003 |
| 2 | .007 | 1.000 | .034 | .003 |
| 3 | .011 | .034 | 1.000 | .005 |
| 4 | -.003 | .003 | .005 | 1.000 |

| Extraction Method: Principal Component Analysis.  Rotation Method: Oblimin with Kaiser Normalization. |
| --- |


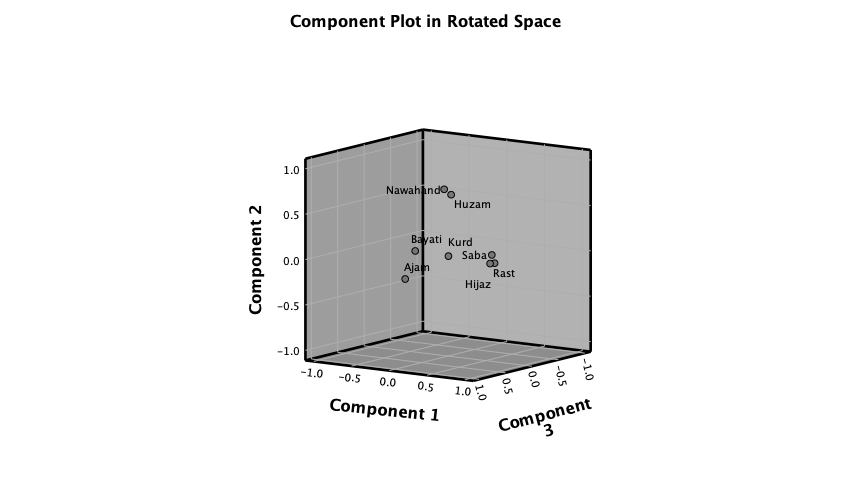


| **Component Score Coefficient Matrix** | | | | |
| --- | --- | --- | --- | --- |
|  | Component | | | |
|  | 1 | 2 | 3 | 4 |
| Kurd | .008 | -.006 | .013 | .980 |
| Saba | .569 | .066 | .017 | -.131 |
| Ajam | -.025 | -.132 | .752 | -.073 |
| Nawahand | -.088 | .694 | -.040 | .028 |
| Hijaz | .560 | -.035 | -.048 | .042 |
| Huzam | .082 | .673 | .076 | -.034 |
| Bayati | .023 | .157 | .644 | .080 |
| Rast | .562 | -.025 | .038 | .076 |

| Extraction Method: Principal Component Analysis.  Rotation Method: Oblimin with Kaiser Normalization.  Component Scores. |
| --- |
